# Divergent HLA variations and heterogeneous expression but recurrent HLA loss-of- heterozygosity and common *HLA-B* and *TAP* transcriptional silencing across advanced pediatric solid cancers

**DOI:** 10.3389/fimmu.2023.1265469

**Published:** 2024-01-22

**Authors:** Wan Ching Lim, Maria Eugenia Marques Da Costa, Karine Godefroy, Eric Jacquet, Loren Gragert, Windy Rondof, Antonin Marchais, Naima Nhiri, Davide Dalfovo, Mathias Viard, Nizar Labaied, Asif M. Khan, Philippe Dessen, Alessandro Romanel, Claudia Pasqualini, Gudrun Schleiermacher, Mary Carrington, Laurence Zitvogel, Jean-Yves Scoazec, Birgit Geoerger, Jerome Salmon

**Affiliations:** ^1^ INSERM U1015, Gustave Roussy Cancer Campus, Université Paris-Saclay, Villejuif, France; ^2^ Bioinformatics Platform, AMMICA, INSERM US23/CNRS UMS3655, Gustave Roussy Cancer Campus, Université Paris-Saclay, Villejuif, France; ^3^ School of Data Sciences, Perdana University, Kuala Lumpur, Malaysia; ^4^ Department of Pathology and Laboratory Medicine, Translational Research Laboratory and Biobank, AMMICA, INSERM US23/CNRS UMS3655, Gustave Roussy Cancer Campus, Université Paris-Saclay, Villejuif, France; ^5^ Institut de Chimie des Substances Naturelles, CNRS UPR2301, Université Paris-Saclay, Gif-sur-Yvette, France; ^6^ Department of Pathology and Laboratory Medicine, Tulane University School of Medicine, New Orleans, LA, United States; ^7^ Department of Cellular, Computational and Integrative Biology (CIBIO), University of Trento, Trento, Italy; ^8^ Frederick National Laboratory for Cancer Research, National Cancer Institute, Frederick, MD, United States; ^9^ Laboratory of Integrative Cancer Immunology, Center for Cancer Research, National Cancer Institute, Bethesda, MD, United States; ^10^ Department of Pediatric and Adolescent Oncology, Gustave Roussy Cancer Campus, Université Paris-Saclay, Villejuif, France; ^11^ INSERM U830, Recherche Translationnelle en Oncologie Pédiatrique (RTOP), and SIREDO Oncology Center (Care, Innovation and Research for Children and AYA with Cancer), PSL Research University, Institut Curie, Paris, France; ^12^ Ragon Institute of Massachusetts General Hospital, MIT and Harvard University, Cambridge, MA, United States

**Keywords:** pediatric cancers, refractory and recurrent solid tumors, immunogenetics, HLA, tumor immunity, immunotherapy

## Abstract

The human leukocyte antigen (HLA) system is a major factor controlling cancer immunosurveillance and response to immunotherapy, yet its status in pediatric cancers remains fragmentary. We determined high-confidence HLA genotypes in 576 children, adolescents and young adults with recurrent/refractory solid tumors from the MOSCATO-01 and MAPPYACTS trials, using normal and tumor whole exome and RNA sequencing data and benchmarked algorithms. There was no evidence for narrowed HLA allelic diversity but discordant homozygosity and allele frequencies across tumor types and subtypes, such as in embryonal and alveolar rhabdomyosarcoma, neuroblastoma *MYCN* and 11q subtypes, and high-grade glioma, and several alleles may represent protective or susceptibility factors to specific pediatric solid cancers. There was a paucity of somatic mutations in HLA and antigen processing and presentation (APP) genes in most tumors, except in cases with mismatch repair deficiency or genetic instability. The prevalence of loss-of-heterozygosity (LOH) ranged from 5.9 to 7.7% in HLA class I and 8.0 to 16.7% in HLA class II genes, but was widely increased in osteosarcoma and glioblastoma (~15-25%), and for *DRB1-DQA1-DQB1* in Ewing sarcoma (~23-28%) and low-grade glioma (~33-50%). HLA class I and HLA-DR antigen expression was assessed in 194 tumors and 44 patient-derived xenografts (PDXs) by immunochemistry, and class I and APP transcript levels quantified in PDXs by RT-qPCR. We confirmed that HLA class I antigen expression is heterogeneous in advanced pediatric solid tumors, with class I loss commonly associated with the transcriptional downregulation of *HLA-B* and transporter associated with antigen processing (*TAP*) genes, whereas class II antigen expression is scarce on tumor cells and occurs on immune infiltrating cells. Patients with tumors expressing sufficient HLA class I and TAP levels such as some glioma, osteosarcoma, Ewing sarcoma and non-rhabdomyosarcoma soft-tissue sarcoma cases may more likely benefit from T cell-based approaches, whereas strategies to upregulate HLA expression, to expand the immunopeptidome, and to target TAP-independent epitopes or possibly LOH might provide novel therapeutic opportunities in others. The consequences of HLA class II expression by immune cells remain to be established. Immunogenetic profiling should be implemented in routine to inform immunotherapy trials for precision medicine of pediatric cancers.

## Introduction

Cancers occurring in children, adolescents and young adults comprise more than 60 tumor types, and are characterized by low mutational rates and recurrent pathognomonic germline or somatic alterations, copy number variations, fusion transcripts, and hijacked enhancers (1–3), exhibiting oncogenic activities in specific cell developmental stages (4–6). It has been estimated that ~8-15% of pediatric cancers develop in the context of a genetic predisposition (7–9). Recently, whole exome (WES) or genome (WGS) and RNA sequencing (RNA-Seq) of paired normal and tumor tissues has provided a comprehensive characterization of cancer predisposition and somatically-mutated genes in pediatric tumors (1, 2, 8, 10–12). However, tumor-immune interactions and immunogenetic factors associated with escape from immunosurveillance remain elusive.

Pediatric solid cancers have been often described as “immune cold” following an early report of a paucity of infiltrating T and dendritic cells and a predominance of macrophages (13), with disappointing responses to immune checkpoint inhibitors (14–16). However, this view has been recently challenged by the description of a marked heterogeneity of T cell infiltration in specific tumor types and subtypes, including neuroblastoma (17–19), rhabdoid tumor (20) and glioma (21). T cell-inflamed tumors are associated with improved overall survival in neuroblastoma (19), and are more frequent in specific low-grade glioma subtypes (pleomorphic xanthoastrocytoma and ganglioglioma) than pilocytic astrocytoma and high-grade glioma (21). A better understanding of tumor-specific T cell responses is of paramount importance for the implementation of effective immunotherapy approaches. Although it is a critical factor in controlling the magnitude, breadth, and specificities of T cell responses, the status of HLA antigens, including their allelic variations and expression patterns, are only partially documented in pediatric cancers.

The HLA complex (6p21.3-22.1) is a highly dense and polymorphic region of the human genome (22). It contains the classical HLA class I (*HLA-A*, *-B*, *-C*) and class II genes (*HLA-DRA*, *-DRB1-DRB3/4/5*, *-DQA1*, *-DQB1*, *-DPA1*, *-DPB1*), encoding highly polymorphic heterodimeric membrane proteins (with class I heavy chains pairing to β2-microglobulin, and class II α and β chains paired together), which associate to peptide fragments (epitopes) recognized by T cell receptors (TCRs) expressed on CD8+ or CD4+ T cells, respectively (23, 24). Nonclassical HLA class I antigens (including HLA-E, -F, -G) have reduced allelic diversity and present epitopes to specific T and natural killer (NK) cell subsets. HLA class I and II antigens are also ligands for T and NK activatory or inhibitory receptors, including NKG2/CD94, killer Ig-like receptors (KIRs), and leukocyte Ig-like receptors (LILRs) (25–27). The HLA class II region contains also genes associated with the class I (*TAP1* and *TAP2*, proteasome 20S subunits (*PSMB*) 8 and 9) and class II antigen presentation pathways (*HLA-DMA*, *-DMB*, *-DOA*, -*DOB*), and the class III region contains genes involved in innate immune responses and inflammation (tumor necrosis factor (*TNF*), lymphotoxins (*LT*) alpha and beta, leukocyte specific transcript 1 (*LST1*), complement factors (*C2*, *BF*, *C4A*, *C4B*), heat shock protein family A (*Hsp70*) members, etc.) (22).

Allelic variations of HLA genes are crucial determinants of the positive and negative selection of the T cell repertoire, the interindividual variability of immune responses to self and foreign proteins, and are linked to the susceptibility or resistance to infectious diseases, autoimmunity, and cancers (23, 28–30). It has been long recognized that major histocompatibility complex (MHC) genotypes select Th1/Th2 polarization (31) and drive the functional diversification of cytotoxic T cell immunity (32). It is now well documented how biophysical properties of peptide-MHC complexes control TCR selection, T cell proliferation and differentiation, and disease outcome (24, 33). Protective and susceptible HLA associations have been reported in adult solid cancers with an infectious etiology, including cervical, head and neck, nasopharyngeal, liver, and gastric cancers (34), as well as in lung cancer (35, 36), and are widely assumed to reflect the capacity to present pathogen- or neoantigen-derived epitopes and to elicit favorable, inefficient, or even protumoral immune responses. HLA class I genotypes and homozygosity restrict the oncogenic mutational landscape in adult solid tumors (37), and HLA class II-restricted cancer driver mutations are negatively selected (38). Recently, HLA genotypes have been shown to influence responses to immune checkpoint inhibitors (ICIs) in adults with advanced solid cancers (39–42). *HLA-B44* and *-B62* supertypes are associated with extended survival and poor outcome, respectively, in ICI-treated patients with melanoma (39), and *HLA-B44* is associated with ICI benefit in patients with non-small cell lung cancer (NSCLC) harboring somatic mutations enriched for B44-motif neoepitopes (41). HLA-A*03 is associated with poor overall survival and shorter progression-free survival in ICI-treated patients across several tumor types, including melanoma, NSCLC, bladder cancer, renal cell carcinoma, and glioma (42). On the other hand, downregulation or loss of HLA class I antigen expression through reversible/”soft” (transcriptional silencing) or irreversible/”hard” genetic defects (somatic alterations) are prominent mechanisms of immune escape and progression reported in adult cancers (43–51).

Early studies of HLA class I expression in specific pediatric solid tumors and cell lines, by immunoflorescence (IF) or immunochemistry (IHC) on snap-frozen or formalin-fixed, paraffin-embedded tumor specimens using various antibodies (such as W6/32, HC-10, HCA2, or EMR8-5), have reported the frequent loss or downregulation of HLA class I antigens in undifferentiated blastema cells of nephroblastoma (52), rhabdomyosarcoma and other soft-tissue sarcomas (53), osteosarcoma (53, 54), neuroblastoma (55), Ewing sarcoma (56, 57), and medulloblastoma (58). Overall, reported frequencies of altered HLA class I immunoreactivity ranged from 52% in primary osteosarcoma to 100% in neuroblastoma (reviewed in (59)). HLA class II expression was not detected in nephroblastoma (52), neuroblastoma (55), and Ewing sarcoma cell lines due to lack of class II transactivator (CIITA) expression (56), and was heterogeneous in osteosarcoma (53). In the context of a program to explore the immune contexture and landscape of HLA-restricted epitopes from candidate tumor antigens in recurrent/refractory pediatric solid cancers, we characterized the HLA genotypes in patients enrolled in the institutional MOSCATO-01 (60) and European MAPPYACTS molecular profiling trials (61), together with the comparative analysis of HLA class I and HLA-DR immunoreactivity and landscape of somatic HLA alterations across solid tumor types and subtypes. Furthermore, we investigated the correlation between HLA class I antigen immunoreactivity and locus-specific transcriptional levels in patient-matched PDX models.

## Results

### HLA allelic diversity and genetic ancestry in the pediatric solid cancer cohort

Normal (WES) and tumor (WES, RNA-Seq) genomic data from 576 patients with recurrent or refractory pediatric solid cancers previously enrolled in MOSCATO-01 (60) and MAPPYACTS (61) were analyzed. The study comprised 11 disease cohorts, including 262 patients with soft-tissue and bone sarcomas (rhabdomyosarcoma; non-rhabdomyosarcoma soft-tissue sarcoma (NRSTS); osteosarcoma; Ewing sarcoma), 141 patients with other extracranial solid tumors (neuroblastoma; nephroblastoma; carcinoma), and 173 patients with central nervous system (CNS) tumors (low-grade glioma (LGG); high-grade glioma (HGG); medulloblastoma; ependymoma) (**Table 1**; **Supplementary Table S1**). Rhabdomyosarcoma and neuroblastoma were stratified into known molecular subtypes: embryonal/fusion-negative (eRMS) and alveolar/fusion-positive rhabdomyosarcoma (aRMS), and neuroblastoma *MYCN*-non amplified (MYCN-NA), *MYCN*-amplified (MYCN-A), 11q wild type (11qWT), and 11q-deleted (11qLOH). HGG were grouped as glioblastoma (GBM) or others (“non-GBM”). All patients had both normal and tumor WES. Tumor RNA-Seq was available for 520 (90.3%) of cases, and 415 of them (80%) had a RIN ≥ 5 (**Table 1**).

**Table 1:** No. of pediatric and young adult cancer patients from the MOSCATO-01 and MAPPYACTS trials included for HLA allelic inference.

Tumor type / subtype^1^	Age mean (range)	Total	Male	Female	Primary	Metastasis	Secondary cancer	With tumor RNA-Seq	
								Total^2^	Contributive^3^
SARCOMAS									
Rhabdomysosarcoma	10.8 (0.8 - 22.8)	68	39	29	28	40		62	47
Embryonal	8.2 (0.8 - 18.1)	34	23	11	18	16		30	23
Alveolar	13.3 (3.5 - 22.8)	34	16	18	10	24		32	24
Osteosarcoma	15.4 (5.0 - 30.8)	72	47	25	7	64	1	68	56
Ewing sarcoma	14.6 (1.7 - 23.8)	61	39	22	12	49		55	46
NRSTS	12.9 (0.5 - 31.8)	61	40	21	27	33	1	56	46
**Total SARC**	**13.4 (0.5 - 31.8)**	**262**	**165**	**97**	**74**	**186**	**2**	**241**	**195**
OTHER EXTRACRANIAL SOLID TUMORS									
Neuroblastoma	7.9 (0.7 - 32.9)	90	51	39	21	69		76	54
*MYCN*-non amplified	8.3 (0.7 - 32.9)	75	42	33	18	57		61	43
*MYCN*-amplified	5.8 (1.4 - 12.5)	15	9	6	3	12		15	11
11qWT	8.1 (0.7 - 25.0)	67	38	29	14	53		56	37
11qLOH	7.5 (2.1 - 32.9)	23	13	10	7	16		20	17
Nephroblastoma	8.6 (1.6 - 18.8)	26	14	12		26		22	20
Carcinoma	14.1 (3.4 - 21.8)	25	11	14	7	18		23	21
**Total Others**	**9.1 (0.7 - 32.9)**	**141**	**76**	**65**	**28**	**113**		**121**	**95**
CNS TUMORS									
Low-grade glioma	11.0 (1.0 - 25.6)	24	12	12	21	3		19	14
High-grade glioma	13.1 (2.1 - 21.3)	63	35	28	39	19	5	60	44
Glioblastoma	12.9 (3.1 - 19.2)	26	16	10	16	6	4	24	19
Others	13.2 (2.1 - 21.3)	37	19	18	23	13	1	36	25
Medulloblastoma	10.7 (3.2 - 27.5)	51	35	16	19	32		45	37
Ependymoma	9.7 (0.9 - 34.5)	35	22	13	21	14		34	30
**Total CNS**	**11.4 (0.9 - 34.5)**	**173**	**104**	**69**	**100**	**68**	**5**	**158**	**125**
**Total**	**11.8 (0.5 - 34.5)**	**576**	**345**	**231**	**202**	**367**	**7**	**520**	**415**

^1^ NRSTS, non-rhabdomyosarcoma soft-tissue sarcoma.

^2^ No. of patients with at least one tumor RNA-Seq analyzed.

^3^ Tumor RNA-Seq were considered as contributive based on evaluation by the molecular tumor board, and RIN ≥ 5.Values shown in bold indicate the total No. of specimens for the main tumor entities, and the overall total No.

**Figure 1:**
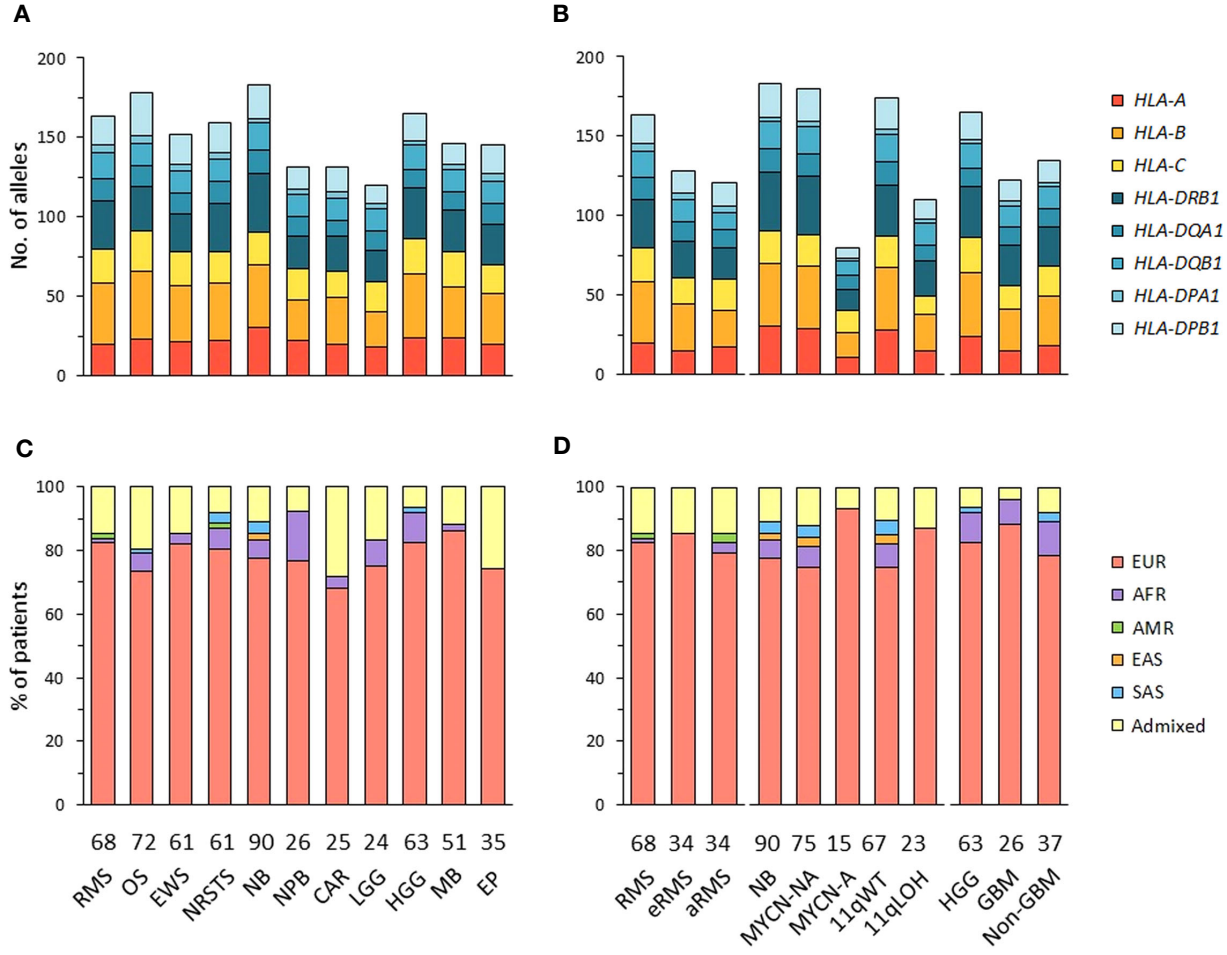
HLA class I and II allelic diversity and genetic ancestry in patients with advanced pediatric solid cancers. **(A, B)** Numbers of HLA class I and II alleles detected in patients with specific tumor types **(A)**, and stratified by molecular subtypes for RMS (eRMS, aRMS), neuroblastoma (MYCN-NA, MYCN-A, 11qWT, 11qLOH), and HGG (GBM, non-GBM) **(B)**. **(C, D)** Predominant genetic ancestry fractions (≥ 70%) of patients with specific tumor types **(C)** and subtypes **(D)**, as determined using EthSEQ (67, 68) based on reference superpopulations (EUR, European; AFR, African; AMR, Native/Latin American; EAS, East Asian; SAS, South Asian). Patients with no predominant genetic ancestry fraction were classified as admixed. The number of patients in each cohort is indicated at the bottom of the corresponding bar charts. RMS, rhabdomyosarcoma; eRMS, embryonal/fusion negative RMS; aRMS, alveolar/fusion positive RMS; OS, osteosarcoma; EWS, Ewing sarcoma; NRSTS, non-rhabdomyosarcoma soft-tissue sarcoma; NB, neuroblastoma; NPB, nephroblastoma; CAR, carcinoma; LGG, low-grade glioma; HGG, high-grade glioma; GBM, glioblastoma; MB, medulloblastoma; EP, ependymoma.

The alleles of eight HLA class I (*HLA-A*, *-B*, *-C*) and class II genes (*HLA-DRB1*, *-DQA1*, *-DQB1*, *-DPA1*, -*DPB1*) were initially inferred from normal WES using HLA-HD (62). To assess possible somatic HLA loss, we also performed HLA typing from tumor WES using HLA-HD, and tumor RNA-Seq using HLA-HD and HLAProfiler (63). In cases with discordant results, HLA typing was repeated from normal WES using Optitype (64) for HLA class I genes, and xHLA (65) and HISAT (66) for HLA class I and II genes. Benchmarking studies demonstrated that combining these algorithms achieves high confidence HLA typing accuracy (**Supplementary Table S2**). Consensus genotypes were successfully established for *HLA-A*, *-B*, *-C* and *-DPA1* in all patients and *HLA-DRB1*, *-DQA1*, *-DQB1*, *-DPB1* in 99.8 to 98.1% of cases. Extended genotypes were established for all eight HLA loci in 560 out of 576 (97.2%) patients, and the genotypes confirmed from tumor samples in 494 of 520 (95%) cases with available tumor RNA-Seq. Overall, 155 HLA class I and 123 HLA class II alleles were identified in the 11 tumor cohorts, with no apparent narrowing of HLA allelic diversity associated with any tumor type or subtype (**Figures 1A, B**; **Supplementary Tables S3A-C**, **Figure S1**).

The genetic ancestry of patients was determined from normal WES using the SNP-based EthSEQ method (67, 68), which accurately defined ancestry fractions in > 99% of patients (*n* = 10,678) from The Cancer Genome Atlas (TCGA) in comparison with a consensus established by combining EthSEQ and four other SNP- and/or WES-based methods (69). The ancestry fractions were computed based on a reference model built using data from the 1000 Genomes Project covering the five major superpopulations (AFR, African; AMR, Native/Latin American; EAS, East Asian; EUR, European; SAS, South Asian) (**Supplementary Figures S2A-L**). Using an ancestry fraction threshold of ≥ 70% for superpopulation assignment, 455 out of 576 (79%) patients showed a predominant EUR ancestry, 30 (5.2%) were AFR, seven (1.2%) were SAS, and 80 (13.9%) patients with no ancestry fraction above the threshold were classified as admixed. Highest frequencies of admixed patients were observed in the carcinoma (28%), ependymoma (25.7%) and osteosarcoma cohorts (19.4%), while the nephroblastoma cohort had the highest frequency of patients with a predominant AFR fraction (15.4%). EUR patients were slightly more frequently observed in neuroblastoma MYCN-A (93.3%) than in MYCN-NA (74.7%) cases (**Figures 1C, D**; **Supplementary Table S4**). Nine tumor cohorts (excluding carcinoma and LGG) comprised ≥ 20 EUR patients, and were further analyzed to infer HLA haplotypes, homozygosity frequencies, and candidate allelic associations.

### HLA haplotypes in EUR patients with pediatric solid cancers

**Figure 2:**
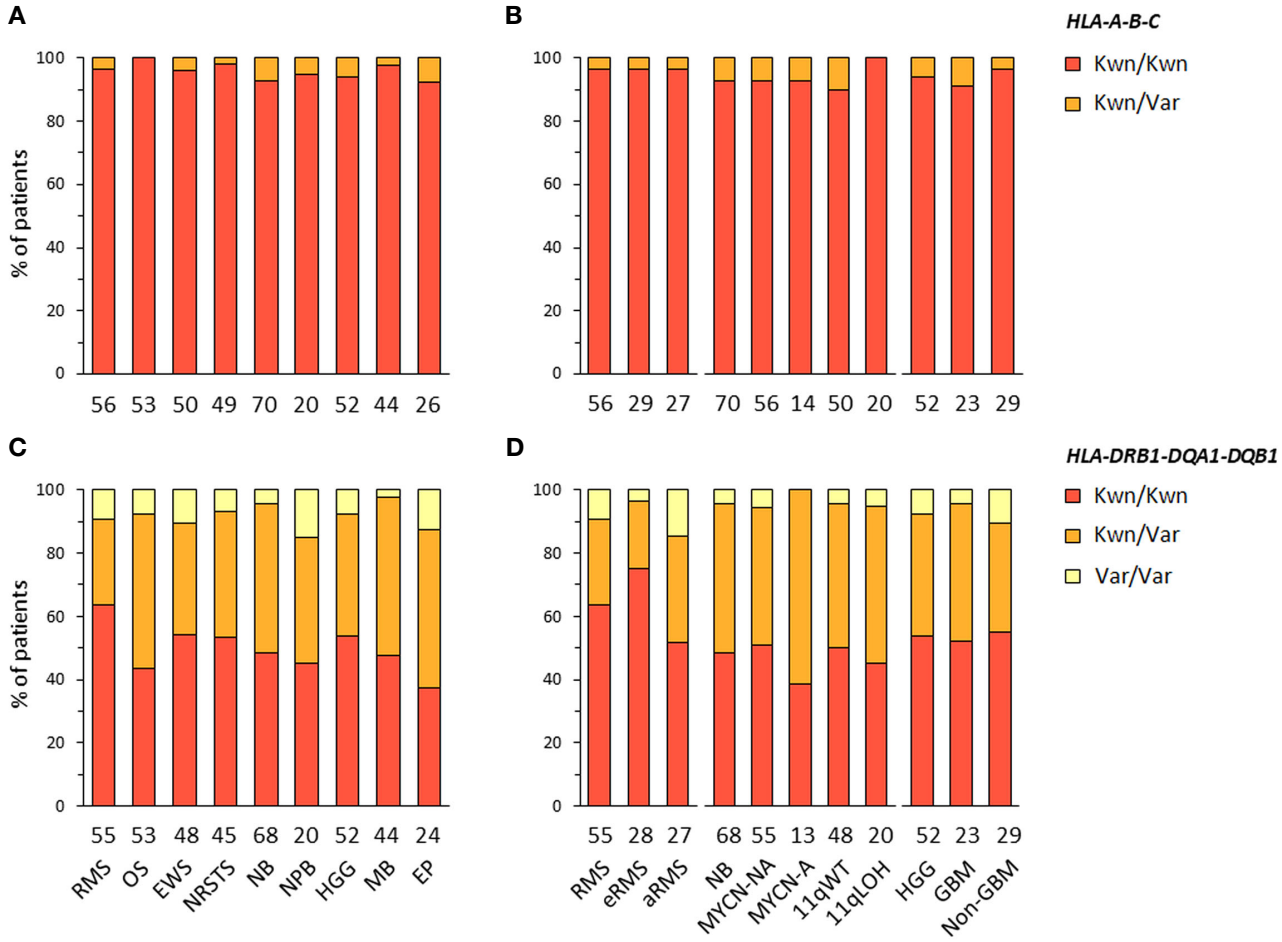
HLA class I and II haplotype combinations in EUR patients with advanced pediatric solid cancers. Inferred *HLA-A-B-C* **(A, B)** and *HLA-DRB1-DQA1-DQB1* haplotypes **(C, D)** were classified as known (Kwn) or variant (Var) in comparison to reference HLA haplotypes reported in EUR controls from the NMDP and Be The Match^®^ repository (70, 71).

*HLA-A-B-C* and *-DRB1-DQA1-DQB1* haplotype combinations were inferred in EUR patients from their corresponding genotypes using an expectation-maximization (EM) algorithm and the Be The Match^®^ registry from the United States National Marrow Donor Program (NMDP), as previously described (70). For *HLA-DRB1-DQA1-DQB1* haplotypes, we also used reference haplotypes (*n* = 75) originally reported in EUR individuals from the NMDP (71). For each patient, the most likely haplotypes were classified as known, variants, or unknown if they were identical to reference haplotypes, differed only by one allele of a single gene at the 4-digit resolution, or did not correspond to any reported haplotype, respectively. Haplotypes containing allele(s) within G groups in the HLA nomenclature, as compared to the closest references, were considered as variants. For *HLA-A-B-C*, we identified 346 haplotypes in 420 EUR patients with consensus genotypes, corresponding to 336 (97.1%) known and 10 (2.9%) variant haplotypes. For *HLA-DRB1-DQA1-DQB1*, congruent results were obtained using the two reference datasets (70, 71), with 73 haplotypes identified in 409 EUR patients with consensus genotypes, corresponding to 35 (47.9%) known and 38 (52.1%) variant haplotypes (**Figures 2A–D**; **Supplementary Tables S5-6**). Of note, 11 and 13 variant haplotypes contained DQA1*03:03 and *05:05, which were not discriminated from DQA1*03:01 and *05:01 in Klitz et al. (71) and are included in G groups (**Supplementary Tables S6B**). The high concordance between HLA genotypes established from next-generation sequencing (NGS) data of patients and known or variant haplotypes demonstrates the accuracy of the typing consensus method used therein.

**Figure 3:**
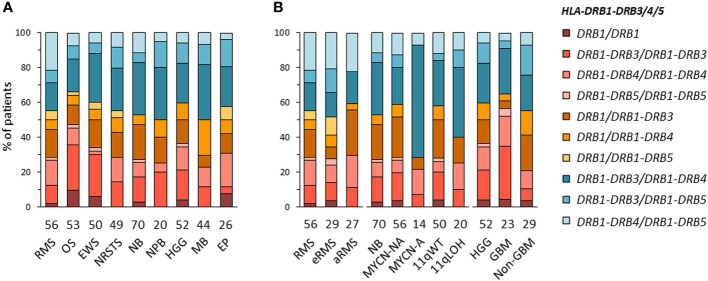
*HLA-DRB1-DRB3/4/5* haplotype combinations in EUR patients with specific solid tumor types **(A)** and subtypes **(B)**. *HLA-DRB1-DRB3/4/5* haplotypes were inferred according to known combinations of *DRB1* with *DRB3*, *DRB4* and/or *DRB5* genes (72–74).

*HLA-DRB1-DRB3/4/5* haplotypes were determined in EUR patients according to the linkage disequilibrium between *DRB1* and *DRB3*, *DRB4* and/or *DRB5* alleles in the corresponding haplotype groups: *DRB1* (DR1, DR8), *DRB1-DRB3* (DR52), *DRB1-DRB4* (DR53), or *DRB1-DRB5* (DR51) (72–74) (**Supplementary Table S7A**). Of note, DRB4*01:03N is a null allele (with a splice site mutation in intron 1), and cannot be discriminated from the *01:03 allele using HLA-HD or HLAProfiler. Overall, *DRB1-DRB3/4/5* haplotypes were resolved from the corresponding genotypes in all 420 EUR patients with consensus typing. The most common haplotype combinations were *DRB1-DRB3/DRB1-DRB4* (~25% of cases), followed by *DRB1-DRB3/DRB1-DRB3* and *DRB1/DRB1-DRB3* (~15% of cases), but there were notable differences among tumor types and subtypes. For instance, the *DRB1-DRB3/DRB1-DRB3* genotype was found in 14 out of 53 (26.4%) patients with osteosarcoma but only in one out of 26 (3.8%) ependymoma; it was also found in seven out of 23 (30.4%) GBM but only in two out of 29 (6.9%) other HGG (“non-GBM”). The *DRB1/DRB1-DRB3* genotype occurred in seven out of 27 (25.9%) patients with aRMS, but only two out of 29 (6.9%) patients with eRMS. Interestingly, about half (48.1%) of aRMS cases had either *DRB1/DRB1-DRB3* or *DRB1-DRB4/DRB1-DRB5*. In neuroblastoma, the *DRB1-DRB3/DRB1-DRB4* genotype was found in nine out of 14 (64.3%) MYCN-A and eight out of 20 (40%) 11qLOH cases but only 12 out of 56 (21.4%) MYCN-NA and 13 out of 50 (26%) 11qWT cases, respectively (**Figures 3A, B**; **Supplementary Tables S7B, C**). Therefore, specific *DRB1-DRB3/4/5* genotypes occur with heterogeneous frequencies in patients with distinct pediatric solid tumors, suggesting *HLA-DR*-dependent associations.

### HLA homozygosity frequencies in EUR patients with pediatric solid cancers

**Figure 4:**
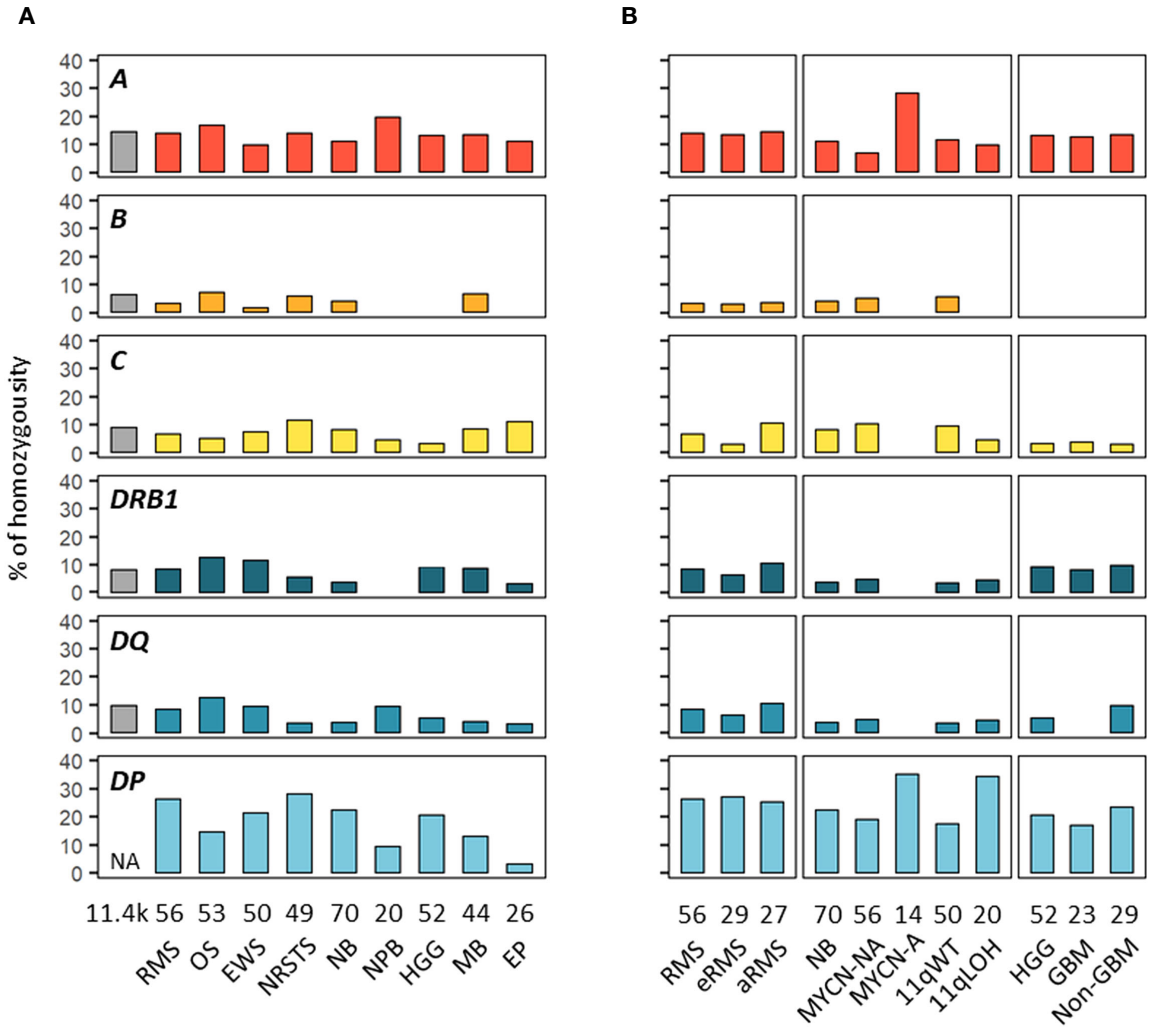
HLA homozygosity frequencies in EUR patients with advanced pediatric solid cancers. The percentages of patients homozygous for HLA class I and II genes are indicated in cohorts corresponding to specific tumor types **(A)** and subtypes **(B)**. Reference frequencies reported in EUR control individuals (75) are shown on the left (grey bar; NA: not available). The label in the upper left corner of each panel refers to the corresponding HLA gene. *DQ* and *DP* refer to *HLA-DQA1-DQB1* and *HLA-DPA1-DPB1*, respectively.

We next investigated HLA homozygosity frequencies in EUR patients in comparison with values reported in control individuals (75). Among HLA class I genes, homozygosity frequencies ranged from 10% (Ewing sarcoma) to 20% (nephroblastoma) for *HLA-A*, from none (HGG, ependymoma, nephroblastoma) to 7.5% (osteosarcoma) for *HLA-B*, and from 3.8% (HGG) to 12.2% (NRSTS) for *HLA-C*, as compared to 14.8, 6.7 and 9.4% of controls, respectively. Interestingly, in neuroblastoma, *HLA-A* homozygosity was detected in four out of 14 (28.6%) patients with MYCN-A tumors but only in four out of 56 (7.1%) MYCN-NA cases. Homozygosity at two or three HLA class I loci occurred in none (HGG, ependymoma, nephroblastoma) to 11.4% of patients (medulloblastoma, five out of 44 cases) (**Figures 4A, B**; **Supplementary Table S8**). Among HLA class II genes, homozygosity frequencies for *DRB1* and *DQA1-DQB1* were low in nephroblastoma (none) and ependymoma (3.8%, a single case), respectively, but were in the higher range in osteosarcoma (13.2%, seven out of 53 cases), as compared to 8.8 and 10.2% of controls, respectively. In NRSTS, homozygosity was rare for *DQA1-DQB1* (4.1%, two cases) but frequent for *DPA1-DPB1* (28.6%, 14 cases). Discordant homozygosity frequencies were also observed between eRMS and aRMS for *DQB1* (6.9 and 18.5%, respectively) and *DPA1* genes (79.3 and 59.3%). In neuroblastoma, *DPA1-DPB1* homozygosity was more frequent in MYCN-A (35.7%) and 11qLOH cases (35%) than MYCN-NA (19.6%) and 11qWT cases (18%). Strikingly, *DPA1* homozygosity occurred in 13 out of 14 (92.9%) MYCN-A cases, but was not detected for *DRB1*, *DQA1*, and *DQA1-DQB1*. In HGG, homozygosity at *HLA-B* was not observed in both GBM and non-GBM cases but was common at *DPA1-DPB1* (17.4 and 24.1%, respectively), whereas homozygosity frequencies at ≥ three HLA class II loci, *DQA1-DQB1*, and *DQB1* alone were slightly higher in non-GBM (13.8 to 10.3%) than GBM cases (none to 4.3%) (**Figures 4A, B**; **Supplementary Table S8**). Thus, discordant homozygosity patterns occurred at distinct HLA loci across tumor types and subtypes, suggesting that specific immunogenetic factors contribute to tumor development and/or progression.

### HLA allele frequencies in EUR patients with pediatric solid cancers

**Figure 5:**
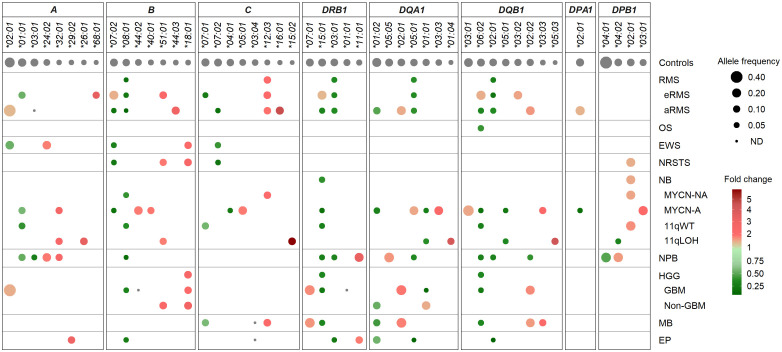
HLA class I and II alleles under- or overrepresented in EUR patients with advanced pediatric solid cancers in comparison with controls. HLA genes and alleles are indicated on the top and ordered by their descending frequencies in EUR control individuals (70, 71, 76, 77). Bubbles are sized according to allele frequencies, and colored by the ratio (fold change) of allele frequencies between patients and controls (underrepresented, green; overrepresented, red).

Despite the limited number of patients per cohort precluding statistical analysis, we investigated HLA allele frequencies in EUR cases to identify candidate alleles which might be positively or negatively associated with pediatric solid tumors, by comparing with mean control frequencies reported for *HLA-A*, *-B* and *-C* (70, 76), and *HLA-DRB1*, *-DQA1*, *-DQB1*, *-DPA1* and *-DPB1* alleles (70, 71, 76, 77) (**Supplementary Tables S9A, B**). Alleles under- or overrepresented in patients were selected based on the following criteria: i) frequency (freq.) ≥ 0.1 in either group and freq. ratios (RA) ≤ 0.5 or ≥ 2 in patients vs. controls; ii) freq. ≥ 0.1 in both groups and RA ≤ 0.67 or ≥ 1.5; and iii) not detected in patients, with freq. in controls ≥ 0.07. Overall, 16 HLA alleles were underrepresented or not detected and 14 alleles were overrepresented in at least one tumor type. Patients with nephroblastoma exhibited the highest allele frequency discordance, with 10 alleles underrepresented (including A*03:01, B*08:01, DRB1*15:01, DQB1*06:02, freq. 0.025 to 0.05, RA 0.23 to 0.38), and five alleles overrepresented (including A*24:02, DRB1*11:01, DQA1*05:05, freq. 0.175 to 0.25, RA 1.82 to 3.54). In contrast, patients with osteosarcoma showed only the DQB1*06:02 allele slightly underrepresented (freq. 0.066, RA 0.5), whereas in patients with Ewing sarcoma, three HLA class I alleles were underrepresented (including B*07:02, C*07:02; freq. 0.05 to 0.07, RA 0.38 to 0.5) and two others overrepresented (A*24:02, B*18:01, freq. 0.17 and 0.11, RA 2.01 and 2.41) (**Figure 5**; **Supplementary Table S9C**).

Several candidate HLA associations were revealed upon stratification into specific tumor subtypes. In rhabdomyosarcoma, patients with eRMS or aRMS showed both common and specific allele frequency discordance in comparison with controls. In both subtypes, several alleles were underrepresented (B*08:01, DRB1*03:01, DQA1*05:01, DQB1*02:01; freq. 0.019 to 0.056, RA 0.17 to 0.48), while the C*12:03 allele was slightly overrepresented (freq. 0.111 to 0.121, RA 2.12 to 2.31). Strikingly, the C*07:01 and *07:02 alleles were specifically underrepresented in eRMS (freq. 0.052, RA 0.34) and aRMS (freq. 0.037, RA 0.26), respectively. Three alleles (B*07:02, DRB1*15:01, DQB1*06:02) were underrepresented in aRMS (freq. 0.037 to 0.056, RA 0.28 to 0.42) but slightly overrepresented in eRMS (freq. 0.207 to 0.214, RA 1.52 to 1.61). The A*03:01 allele was not detected in aRMS but occurred at the expected frequency in eRMS (0.138). Other alleles were specifically overrepresented in eRMS (A*68:01, B*51:01, DQB1*03:02; freq. 0.103 to 0.179, RA 1.70 to 3.63) and aRMS (including B*44:03, C*16:01, DQB1*02:02; freq. 0.148 to 0.185, RA 1.81 to 4.67) (**Figure 5**; **Supplementary Table S9C**).

In neurobastoma, 28 alleles had biased frequencies among MYCN-NA, MYCN-A, 11qWT, and/or 11qLOH cases, but none was commonly under- or overrepresented in all subtypes. Twenty-one and 15 alleles had discordant frequencies between MYCN-NA and MYCN-A cases and 11qWT and 11qLOH cases, respectively. Two alleles were under- (B*08:01) and overrepresented (DPB1*02:01) in MYCN-NA (freq. 0.054 and 0.205, RA 0.49 and 1.69, respectively) and 11qWT (freq. 0.05 and 0.230, RA 0.46 and 1.89, respectively). Three alleles (A*01:01, DRB1*15:01, DQB1*06:02) were underrepresented in both MYCN-A and 11qWT (freq. 0.036 to 0.107, RA 0.26 to 0.66, respectively), and two alleles (DQA1*01:01, DQB1*05:01) were underrepresented in both MYCN-A and 11qLOH (freq. 0.036 to 0.05, RA 0.31 to 0.47). In MYCN-A, several other alleles were under- (B*07:02, C*04:01, DQA1*01:02, DPA1*02:01, freq. 0.036 to 0.077, RA 0.25 to 0.39) or overrepresented (B*44:02, C*05:01, DQA1*05:01, DQA1*03:03, DQB1*03:01, DPB1*03:01, freq. 0.179 to 0.321, RA 1.67 to 2.48). In 11qLOH, another allele was underrepresented (DPB1*04:02, freq. 0.05, RA 0.40), and several others overrepresented (including A*26:01, A*32:01, C*15:02, DQA1*01:04, DQB1*05:03, freq. 0.10 to 0.125, RA 2.71 to 5.77) (**Figure 5**; **Supplementary Table S9C**).

In HGG, 13 HLA alleles showed discordant frequencies, including 11 and 4 alleles in GBM and other HGG cases, respectively. One allele (B*18:01) was overrepresented in both groups (freq. 0.109 to 0.138; RA 2.39 to 3.03). In GBM, 10 alleles were specifically underrepresented (B*08:01, DRB1*15:01, DQA1*01:01, DQB1*06:02) (freq. 0.022 to 0.043, RA 0.20 to 0.40), not detected (B*44:02, DRB1*01:01), or overrepresented (DRB1*07:01, DQA1*02:01, DQB1*02:02; freq. 0.196 to 0.261, RA 1.91 to 2.08), whereas in other HGG, three alleles were under- (DQA1*01:02; freq. 0.121, RA 0.62) or overrepresented (B*51:01, DQA1*01:01; freq. 0.121 to 0.172, RA 1.62 to 2.44). The DQA1*01:01 allele was underrepresented in GBM (freq. 0.022, RA 0.20) but slightly overrepresented in other HGG (freq 0.172, RA 1.62). Patients with medulloblastoma showed marked HLA class II allele frequency discordance, with several alleles under- (DRB1*15:01, DQA1*01:02, DQB1*06:02; freq. 0.057 to 0.102, RA 0.42 to 0.52) or overrepresented (including DRB1*07:01, DQA1*02:01, DQB1*02:02 and *03:03; freq. 0.102 to 0.25, RA 1.78 to 2.37). Furthermore, the C*03:04 and *12:03 alleles were not detected and overrepresented (freq. 0.125, RA 2.39), respectively. Patients with ependymoma showed eight HLA alleles underrepresented (B*08:01, DRB1*03:01, DQA1*01:02 and *05:01, DQB1*02:01, freq. 0.019 to 0.125, RA 0.16 to 0.64), not detected (C*03:04), or slightly overrepresented (A*29:02, DRB1*11:01, freq. 0.115, RA 2.04 to 3.27) (**Figure 5**; **Supplementary Table S9C**).

Overall, several HLA class I and II alleles were underrepresented (B*07:02, B*08:01, C*07:02, DRB1*15:01, DRB1*03:01, DQA1*01:02, DQA1*05:01, DQB1*06:02, DQB1*02:01) or overrepresented (B*51:01, B*18:01, C*12:03) across distinct pediatric solid tumor types or subtypes, whereas others exhibit specific discordant frequencies (including A*03:01, C*07:01 and DQA1*01:01 not detected or underrepresented in aRMS, eRMS and GBM, respectively). Worth stressing, the aforementioned allele variations translated into several candidate haplotype associations. For instance, the A*02:01-B*44:02-C*05:01 haplotype was overrepresented particularly in neuroblastoma MYCN-A, ependymoma and aRMS (freq. 0.143 to 0.093, RA 5.7 to 3.7) (**Supplementary Table S5B**). The DRB1*15:01-DQA1*01:02-DQB1*06:02 haplotype was widely underrepresented, particularly in aRMS, osteosarcoma, neuroblastoma (MYCN-NA, MYCN-A, 11qWT), nephroblastoma, GBM and medulloblastoma (freq 0.038 to 0.073, RA 0.31 to 0.58) but overrepresented in eRMS (freq. 0.214, RA 1.71). The DRB1*03:01-DQA1*05:01-DQB1*02:01 haplotype was underrepresented in eRMS, aRMS, nephroblastoma and ependymoma (freq 0.021 to 0.056, RA 0.18 to 0.48) but overrepresented in neuroblastoma MYCN-A (freq 0.192, RA 1.65). The DRB1*07:01-DQA1*02:01-DQB1*02:02 haplotype was overrepresented in aRMS, GBM and medulloblastoma (freq 0.182 to 0.196, RA 1.84 to 1.98) but underrepresented in nephroblastoma (freq. 0.05, RA 0.51) (**Supplementary Table S6B**). Altogether, these data suggest that specific HLA variations could represent susceptibility or protective factors in the development of pediatric solid tumors.

### HLA class I and HLA-DR antigen expression in advanced pediatric solid tumors

**Figure 6:**
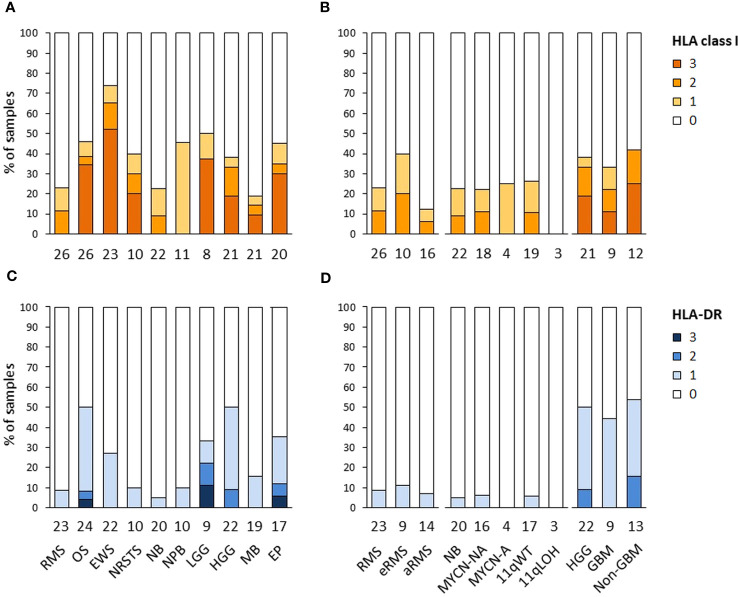
HLA class I and HLA-DR antigen expression in advanced pediatric solid tumors. The percentages of specimens with HLA class I **(A, B)** and HLA-DR **(C, D)** immunoreactivity on tumor cells assessed by IHC were scored using a semi-quantitative scale as follows: 3 (“high”, ≥ 70% tumor cells), 2 (“intermediate”, ≥ 30 to < 70%), 1 (“low”, ≥ 5 to < 30%), and 0 (negative, < 5%). Tumor samples are classified by specific tumor types **(A, C)**, or subtypes for RMS (eRMS, aRMS), neuroblastoma (MYCN-NA, MYCN-A, 11qWT, 11qLOH), and HGG (GBM, non-GBM) **(B, D)**. The numbers of samples are indicated below the corresponding bar charts.

**Figure 7:**
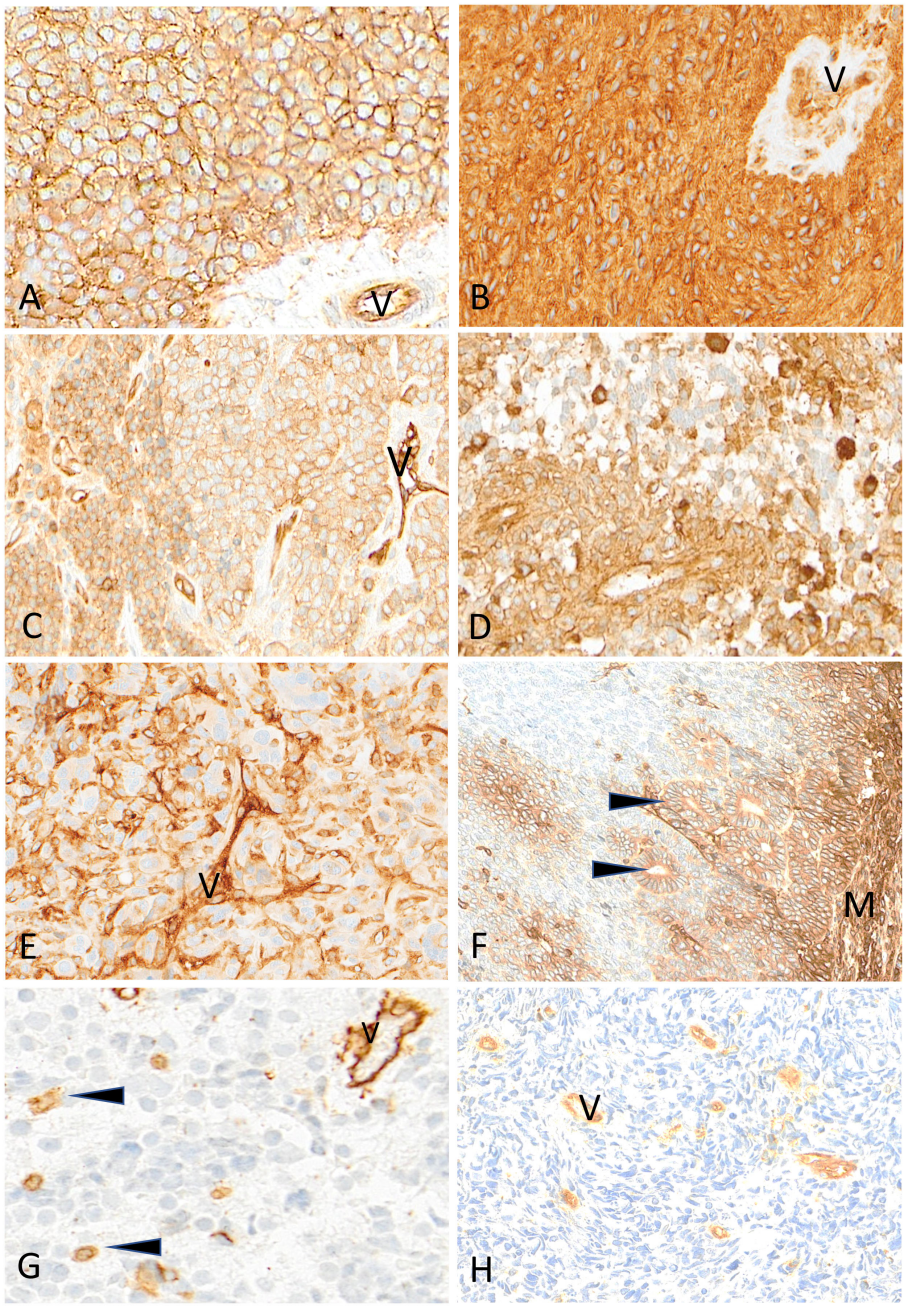
Representative examples of HLA class I immunostaining in advanced pediatric solid tumors. All tumor cells in a case of Ewing sarcoma **(A)** and a case of ependymoma **(B)** are homogeneously and strongly labeled by anti-HLA class I antibody (V, tumor vessel). In **(C)** all tumor cells in this other case of Ewing sarcoma are reactive with anti-HLA class I antibody but with variable apparent levels of intensity (V, tumor vessel as internal control). In a case of HGG **(D)** and in a case of osteosarcoma **(E)**, HLA class I shows a patchy expression in tumor cells (V, tumor vessel; arrowheads, immune cells). In this case of nephroblastoma **(F)**, HLA class I is strongly expressed by mesenchymal cells (M) and tubular epithelial structures (arrowheads) but is undetectable in undifferentiated blastema cells. In a case of neuroblastoma **(G)** and a case of rhabdomyosarcoma **(H)**, HLA class I is undetectable on tumor cells while immune cells (arrowheads) and tumor vessels (V) are labeled. Indirect immunoperoxidase with nuclear counterstaining by hematoxylin. Original magnifications: **(A–C)** x220; **(D)** x280; **(E)** x220; **(F)** x180; **(G)** x320; **(H)** x280.

**Figure 8:**
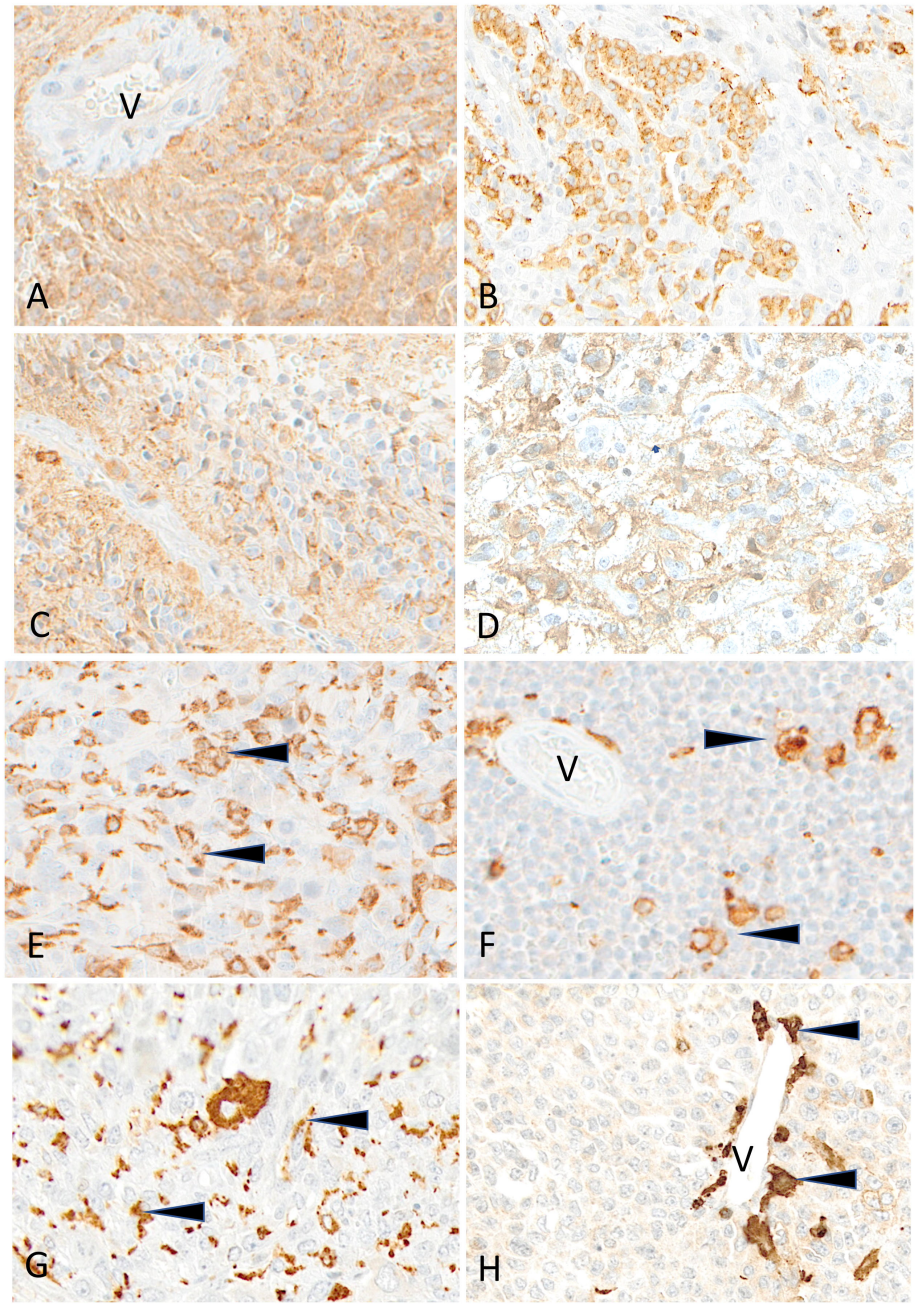
Representative examples of HLA-DR immunostaining in advanced pediatric solid tumors. HLA-DR is homogeneously expressed in a case of ependymoma **(A)** (V, tumor vessel). A patchy expression is visible in cases of osteosarcoma **(B)**, of HGG **(C)** and of LGG **(D)**. HLA-DR is undetectable on tumor cells in cases of osteosarcoma **(E)**, Ewing sarcoma **(F)**, rhabdomyosarcoma **(G)** and neuroblastoma **(H)**; variable numbers of immune cells (arrowheads) are positive (V, tumor vessel). Indirect immunoperoxidase with nuclear counterstaining by hematoxylin. Original magnifications: **(A)** x200; **(B)** x180; **(C, D)** x280; **(E)** x220; **(F)** x250; **(G)** x220; **(H)** x300.

The expression of HLA class I and HLA-DR antigens in tumors was assessed by IHC on formalin-fixed paraffin-embedded tumor specimens using the pan-HLA class I monoclonal antibody EMR8-5 recognizing HLA-A, -B, and -C heavy chains (54), and a HLA-DRβ-specific polyclonal antibody. A total of 188 and 176 specimens were analyzed for HLA class I and HLA-DR immunoreactivity, including sarcomas (*n* = 85 and 79), other extracranial solid tumors (*n* = 33 and 30), and CNS tumors (*n* = 70 and 67), respectively. The percentages of HLA-positive tumor cells were scored using a semi-quantitative scale as follows: 3 (“high”, ≥ 70%), 2 (“intermediate”, ≥ 30 to < 70%), 1 (“low”, ≥ 5 to < 30%), and 0 (negative, < 5%) (**Figures 6A–D**; **Supplementary Tables S10A, B**). Strikingly, high HLA class I antigen expression (score 3) was detected in only 27.1 and 21.4% of sarcoma and CNS tumors, respectively, and none of other extracranial solid tumors. Conversely, complete loss of HLA class I antigen immunoreactivity (score 0) was observed in 69.7, 64.3 and 54.1% of other extracranial solid tumors, CNS tumors, and sarcomas, respectively. Tumor types with elevated frequencies of high HLA class I-positive samples included Ewing sarcoma, osteosarcoma, and ependymoma (52.2 to 30%), while absence of HLA class I antigen immunoreactivity was most frequent in medulloblastoma, rhabdomyosarcoma, and neuroblastoma (81 to 76.9%). HLA class I-positive specimens in nephroblastoma, neuroblastoma and rhabdomyosarcoma tumors (45.5, 22.7 and 23%, respectively) showed only low or heterogeneous HLA class I expression (scores 1 to 2), with no apparent difference among tumor subtypes (eRMS *vs.* aRMS; NB MYCN-A, MYCN-NA, 11qWT *vs.* 11qLOH) (**Figures 6A, B**, **7**). For HLA-DR, high tumor cell expression (score 3) was detected only in single specimens of osteosarcoma, LGG and ependymoma. Low and diffuse or heterogeneous HLA-DR expression (scores 1 to 2) was most frequent in HGG, osteosarcoma, ependymoma and Ewing sarcoma (50 to 27.3%). In contrast, a complete absence of HLA-DR antigen immunoreactivity on tumor cells was observed in 93.3, 73.4 and 65.7% of other extracranial solid tumors, sarcoma and CNS tumors, respectively, and was particularly prominent in neuroblastoma, rhabdomyosarcoma, NRSTS and nephroblastoma (95 to 90%) (**Figures 6C, D**, **8**). Interestingly, in HGG, there was a trend toward lower or negative HLA class I and HLA-DR expression in GBM in comparison with other HGG tumors (**Figures 6B, D**). Altogether, these observations indicate that a majority of advanced pediatric solid tumors exhibit a partial or complete lack of classical HLA class I and an absence of tumor HLA-DR antigen immunoreactivity.

### Paucity of somatic mutations in coding sequences of HLA and APP genes

**Figure 9:**
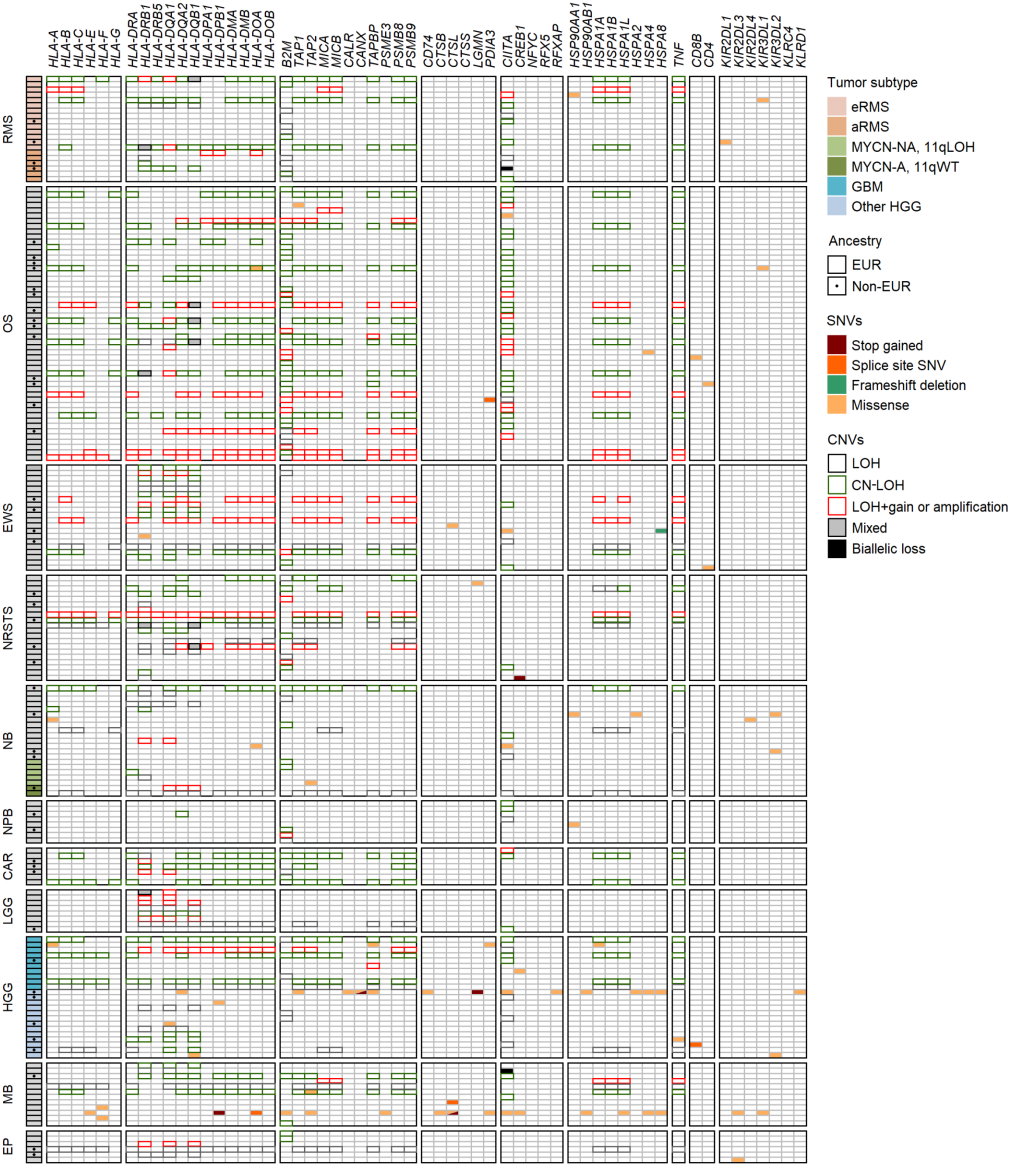
Landscape of SNVs and CNVs in HLA and APP genes in advanced pediatric solid tumors. Mutations detected in the coding sequences of 47 out of 78 selected genes (KEGG pathway hsa04612) are illustrated according to the type of SNVs and their consequence. Tumor subtypes are shown when applicable, and non-EUR patients indicated by a dot. CNVs in the HLA region, *B2M* and *CIITA* genes associated with LOH, LOH+amplification, CN-LOH and/or biallelic losses are indicated by colored boxes.

We next investigated the landscape of somatic single nucleotide variations (SNVs) in 78 HLA and APP genes in solid tumors of 439 patients from the MAPPYACTS cohort, through variant calling of paired tumor and normal WES. Overall, we detected non-silent somatic SNVs within coding sequences (CDS) only in a minority of cases, with 36 tumor specimens (8.2%) harboring between one and 23 mutation(s) in 47 of the tested genes. The highest frequency of mutations was observed in primary HGG (non-GBM) tumors (4 out of 19 cases, 21.1%), as well as metastatic neuroblastoma MYCN-NA 11qWT, medulloblastoma, and osteosarcoma specimens (4 to 7 cases, 14.3 to 12.7%), whereas no mutation was detected in aRMS, neuroblastoma MYCN-A, carcinoma and LGG tumors, and only two nephroblastoma and ependymoma cases harbored single mutations. There was a slight enrichment of mutations in metastases (26 out of 286 cases, 9.1%) in comparison with primary tumors (10 out of 153 cases, 6.5%) (**Figure 9**; **Supplementary Table S11A**). Four HGG, one neuroblastoma and one medulloblastoma specimens, which occurred in the context of mismatch repair deficiency and/or mutations in *TP53*, *NF1*, *H3-3A*, and/or *BRCA2* indicative of elevated genetic instability, harbored between one and up to 23 mutations in 17 genes (**Supplementary Table S11B**). Ten cases of nonsense mutations or frameshift deletions resulted in premature stop codons in *HLA-DPB1* and *cathepsin L* (medulloblastoma), *calnexin* and *legumain* (HGG), *HSPA8* (Ewing sarcoma), and *CREB1* (NRSTS), and in splice site variants in *HLA-DOA* and *cathepsin L* (medulloblastoma), *PDIA3* (osteosarcoma), and *CD8B* (HGG). Only two tumors harbored missense mutations in *HLA-A* CDS, a single one in *B2M*, and five others in *TAP1* and/or *TAP2*, including one medulloblastoma with mutations in both *B2M* and *TAP2*. Interestingly, seven missense mutations in *CIITA*, the master regulator of HLA class II expression, were detected in five tumors, including three mutations in a medullobastoma with constitutional mismatch repair deficiency (**Supplementary Table S12A**). The maximum and median fractions of mutated reads in tumors were 0.59 and 0.35, respectively, indicating that mutations were monoallelic, with the exception of a missense *HSPA4* mutation in one osteosarcoma specimen (0.97). Overall, only two medullobastoma and HGG tumors harbored mutations which can be unambiguously assumed to affect APP (stop codons in *HLA-DPB1* and *calnexin*) (**Figure 9**; **Supplementary Tables S11A, B**). Therefore, SNVs consistent with impaired HLA class I and II presentation pathways are infrequent in advanced pediatric solid tumors, supporting that the common loss of HLA class I expression and scarcity of HLA class II expression must rather involve copy number variations (CNVs), or occur at the regulatory or transcriptional level.

### LOH caused by CNVs are recurrent in HLA class I and II and APP genes

**Figure 10:**
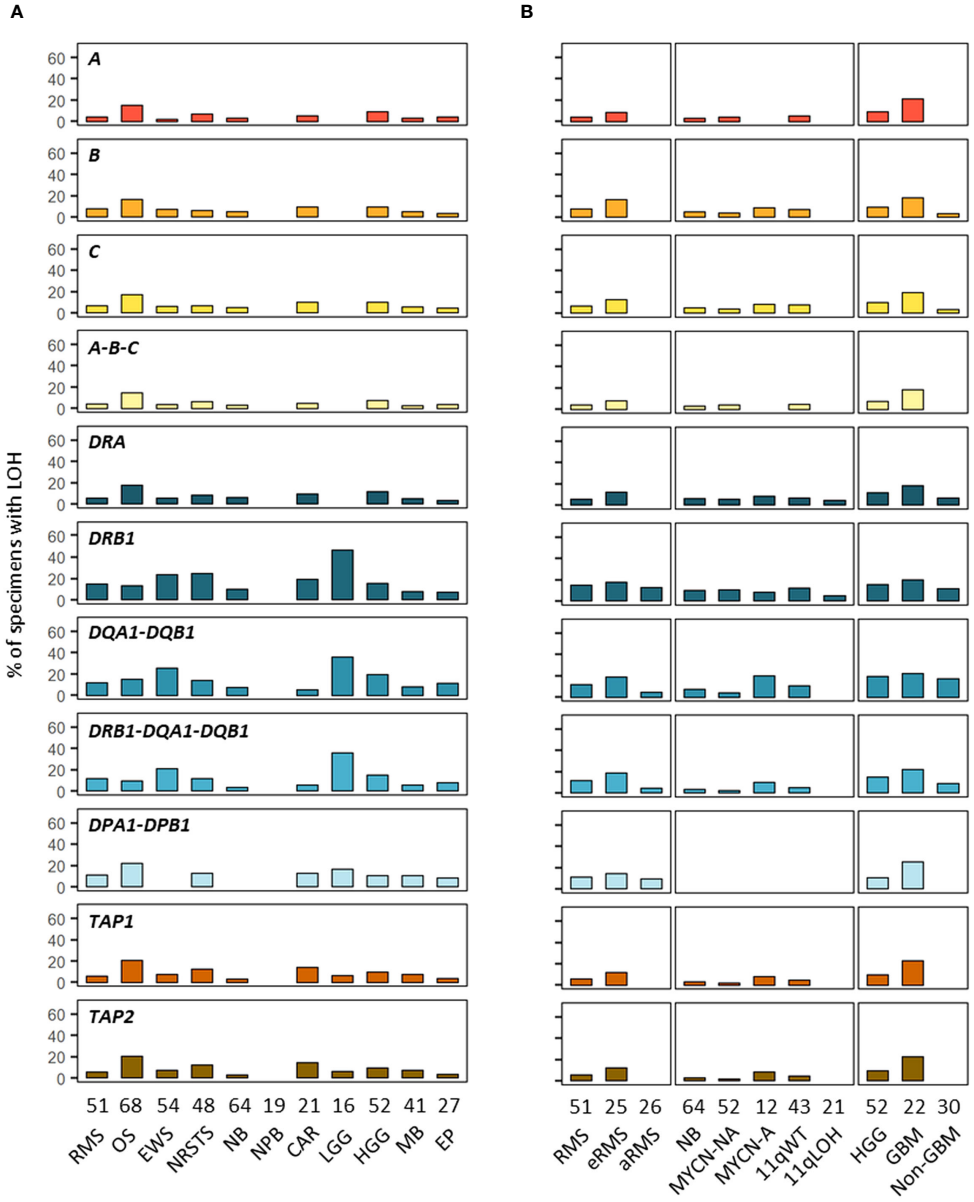
Prevalence of HLA LOH in advanced pediatric solid tumor types **(A)** and subtypes **(B)**. The label in the upper left corner of each panel refers to the corresponding HLA gene(s) or their combination.

In addition to SNVs, tumor HLA alterations can also result from CNVs, eventually leading to HLA and/or APP loss through LOH or copy-neutral (CN)-LOH (43, 44, 46–51). We analyzed CNV data of paired normal and tumor WES generated using the Sequenza pipeline (78) in the context of the MAPPYACTS study for the chromosome arms 6p (HLA region), 15q and 16p (containing the *B2M* and *CIITA* genes, respectively). CNVs with size ≥ 50 kb were retained and categorized as LOH, CN-LOH, and LOH with co-occurring gain or amplification (thereafter collectively referred to as LOH), as well as biallelic loss. Overall, we detected 188, 67 and 59 CNV events in the HLA region, *B2M* and *CIITA* genes, respectively, in 175 out of 461 (38%) tumors. Tumors with CNVs associated with LOH in the HLA region harbored a mean of 1.9 +/- 1.3 CNVs with a median size ~450 kb (max. 32.4 Mb). In contrast, all cases in *B2M* and *CIITA* harbored single CNV events with median sizes of 34.0 and 20.9 Mb, and arm-level 15q and 16p CNVs (≥ 90% of arm length) were detected in eight (11.9%) and nine tumors (15.3%), respectively (**Figure 9**; **Supplementary Tables S12A-C**). The frequency of cases with HLA CNVs was comparable in all primary tumors and metastases (38.7 and 37.6%), but slightly increased in sarcoma metastases (52.2%). Altogether, CNVs associated with HLA loss had a median gene content of 25 annotated elements. Excluding cases with germline homozygous HLA alleles, 34 (7.7%), 32 (7.5%) and 24 (5.9%) tumors exhibited LOH affecting *HLA-B*, *-C* and *-A* genes, respectively, and 26 (5.9%) tumors had LOH affecting all three genes. Extended HLA class I LOH was detected in nine (14.5%) osteosarcoma and four (18.2%) GBM, but not in aRMS, neuroblastoma MYCN-A and 11qLOH, nephroblastoma, and LGG tumors. Furthermore, 49 (10.6%) tumors had LOH affecting APP gene(s) in the HLA region (*TAP1*/2, *TAPBP*, *PSMB8*/*9*, *HSPA1A-L*), including 14 (20.6%) osteosarcoma and five (22.7%) GBM cases with LOH in both TAP1 and TAP2. HLA class I and TAP1/2 LOH co-occurred in 31 out of 47 tumors, indicating a broad immunoediting process in the HLA-I presentation pathway. In addition, *B2M* LOH (without CN-LOH, LOH+gain or amplification) was detected in 18 (3.9%) tumors, including six (11.8%) rhabdomyosarcoma cases (**Figures 9**, **10**; **Supplementary Table S12C**). Among HLA class II genes, LOH events were recurrently detected across multiple cancer types, with 67 (16.7%), 65 (15.6%) and 62 (15.5%) tumors exhibiting LOH in *HLA-DQA1, -DRB1*, and *-DQB1* genes, respectively; 42 (11.0%) cases had LOH in all three genes, including nine (20.9%) Ewing sarcoma, five (35.7%) LGG, and four (22.2%) GBM tumors. Notably, *DRB1-DQA1-DQB1* LOH was detected in four out of 21 (19.0%) eRMS but only one out of 21 (4.8%) aRMS specimens. On the other hand, there was a slightly elevated prevalence of LOH in the *HLA-DP* region in osteosarcoma, with 12 (21.8%) and five (21.7%) cases in *HLA-DPB1* and both *HLA-DPA1-DPB1*, respectively. In addition, 13 (2.8%) tumors had LOH in the *CIITA* gene, including two cases (aRMS and medullobastoma) with biallelic loss (**Figures 9**, **10**; **Supplementary Table S12C**). Overall, 23.5 to 28.0% of eRMS, osteosarcoma and GBM tumors exhibited LOH in at least one gene of the HLA class I pathway (*HLA-A*, *-B*, *-C*, *TAP1, TAP2* and/or *B2M*), and 25.9 to 43.8% of osteosarcoma, Ewing sarcoma, NRSTS, LGG and HGG (non-GBM) tumors exhibited LOH in at least one gene of the HLA class II pathway (*HLA-DRB1*, -*DQA1*, *-DQB1*, *-DPA1*, *-DPB1* and/or *CIITA*). Thirteen (19.1%) osteosarcoma and four (18.2%) GBM tumors exhibited LOH events in both HLA class I and II pathways (**Supplementary Table S12C**). Although our analysis did not address focal CNVs (< 50 kb) and likely underestimates LOH frequencies, these data suggest that CNVs are more prevalent than SNVs in the HLA region and are associated with recurrent HLA class I and II LOH in advanced pediatric solid cancers.

### HLA class I genotypes, antigen expression and transcriptional levels in PDX models

**Figure 11:**
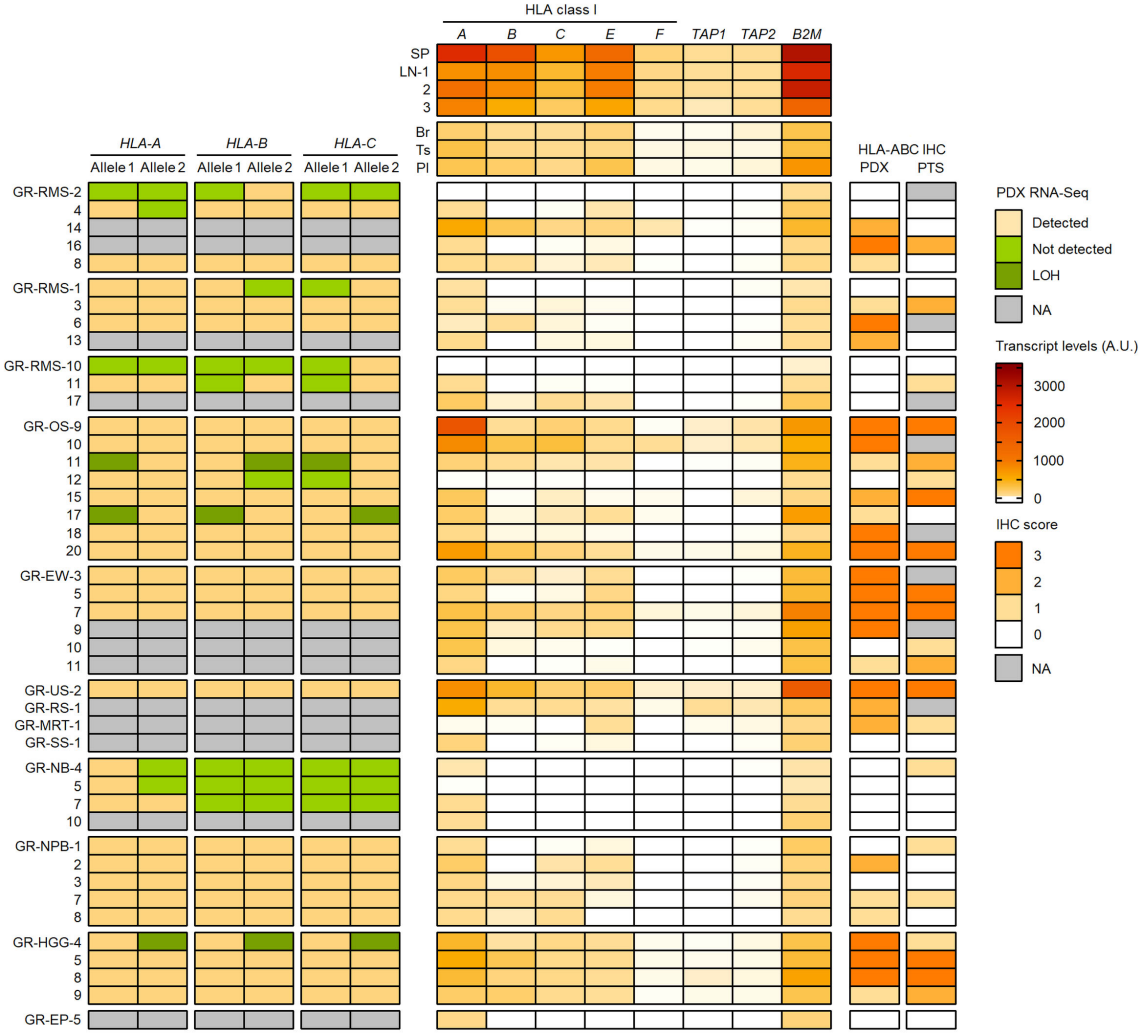
Detection of HLA class I alleles, quantification of HLA and APP transcripts, and IHC scores in PDX models in comparison with primary tumor samples. HLA class I (*A–C*) alleles detected (light brown) or not (green) from PDX WES and RNA-Seq in comparison with normal and patient tumor NGS data are illustrated on the left panel. The upper, middle and lower RMS groups correspond to eRMS, aRMS, and other RMS PDXs, respectively. Patterns consistent with HLA class I LOH are indicated in dark green. Relative transcript levels (arbitrary units, A.U. x100) of HLA class I classical (*A–C*) and nonclassical (*E*, *F*) genes, *TAP1*, *TAP2* and *B2M* as quantified by RT-qPCR (Taqman assay) in selected normal tissues (top) and PDXs (bottom) are shown on the middle panel. HLA class I IHC scores (using the EMR8-5 mAb) in comparison between PDXs and the corresponding primary tumor specimens are indicated on the right panel.

To better understand the HLA class I status in pediatric solid cancers, we further characterized the HLA class I genotypes, antigen expression and transcriptional levels in 44 PDX models established in the context of an ancillary study of MAPPYACTS (79). The propagation of PDXs in immunodeficient mice is accompanied by depletion of human immune cells from the tumor microenvironment (TME), thus avoiding confounding effects on HLA typing and transcript quantification. We established HLA class I genotypes from PDX WES and RNA-Seq in comparison with normal and primary tumor samples (PTS), together with the quantification of HLA class I tumor immunoreactivity by IHC and HLA transcripts by RT-qPCR (Taqman assay). In 20 PDX models, HLA typing congruently detected two alleles for *HLA-A*, *-B* and *-C* identical to those identified from normal and PTS, including all three Ewing sarcoma, five nephroblastoma, and three out of four HGG PDXs. In contrast, no *HLA-B* and *-C* allele was detected from all three neuroblastoma PDXs with available RNA-Seq and only one *HLA-A* allele was detected in two of them, suggesting low transcript levels. Furthermore, no *HLA-A* and only a single *HLA-B* or *-C* allele was detected from RNA-Seq of two rhabdomyosarcoma PDXs (GR-RMS-2 and -10), and single *HLA-B* and *-C* alleles were detected in two others (GR-RMS-1 and -11). Of note, HLA typing from PDX WES and RNA-Seq confirmed the LOH events affecting *HLA-A*, *-B* and *-C* genes identified in the corresponding patient tumors in two out of eight osteosarcoma (GR-OS-11 and GR-OS-17) and one out of four HGG PDXs (GR-HGG-4, GBM) (**Figure 11**; **Supplementary Table S13**).

Overall, there was a high concordance between HLA class I IHC scores in PDX models and patient-matched PTS. Among 36 PTS and PDX pairs with IHC, 17 pairs showed identical IHC scores and 10 others showed higher scores in PTS. High IHC immunoreactivity (score 3) was detected in three out of four HGG (one GBM and two pleomorphic anaplastic xantho-astrocytomas), four out of six Ewing sarcoma, and four out of eight osteosarcoma PDXs. Conversely, an absence, low and focal, or heterogeneous HLA class I expression (score 0-2) was observed in all four neuroblastoma, five nephroblastoma, and 10 out of 12 rhabdomyosarcoma PDXs. Three eRMS and two nephroblastoma PDXs showed higher IHC scores than the PTS, possibly due to intratumoral heterogeneity, and/or immune pressure in patients alleviated upon PDX propagation. HLA class I LOH was associated with a partial loss of IHC immunoreactivity (score 1) in the two osteosarcoma but not GBM PDXs (**Figure 11**; **Supplementary Table S13**).

We quantified the relative transcript levels of nine genes by RT-qPCR (Taqman assay) in both normal tissues and PDXs, including HLA class I classical (*HLA-A*, *-B*, *-C*) and nonclassical (*HLA-E*, *-F*, *-G*) genes, *TAP1*, *TAP2*, and *B2M*. Normal human tissues used as references included spleen (SP, pool from 15 donors), lymph nodes (LNs, three donors), brain (Br), testis (Ts) and placenta (Pl) (pools from five donors each), corresponding to tissues with high (SP, LN) or low (Br, Ts, Pl) *HLA-A*, *-B* and *-C* transcript levels (80) (**Supplementary Tables S14A, B**). In normal tissues, the highest transcript levels were detected in SP followed by LNs, with mean SP/LNs ratio from 2.7 (*HLA-A* and *-B*) to 1.4 (*B2M*), whereas the lowest levels were detected in Br (*HLA-A*, *-B*, *-C*, *-F*, *TAP1*, *B2M*) and Ts (*HLA-E* and *TAP2*), with Br/SP ratio of 0.02 to 0.12 and Ts/SP ratio of 0.06 to 0.16 (**Figure 11**; **Supplementary Table S15**). In PDXs, there was a recurrent pattern of low to negligible HLA class I, *TAP1/2* and *B2M* transcript levels, in most cases below the lowest values of normal tissues (Br, Ts) and correlating with the absence or low HLA class I immunoreactivity (IHC score 0-1). Indeed, all four neuroblastoma, five nephroblastoma, and 11 out of 12 rhabdomyosarcoma PDXs showed relative transcript levels below or within the same range as the lowest values for all tested genes. Conversely, three HGG, four Ewing sarcoma, and four osteosarcoma PDXs with high HLA class I immunoreactivity (score 3) displayed elevated *HLA-A* and *B2M* transcript levels in most cases, whereas *HLA-B* or both *HLA-B* and *-C* transcript levels were below the lowest value (Br) in one (HGG) to three (Ewing sarcoma) PDXs. Finally, HLA and APP transcript levels were heterogeneous in NRSTS, with only one undifferentiated sarcoma with a *ETV6::NTRK3* fusion displaying high transcript levels and IHC score, and were negligible in a single EP PDX. Overall, 32 out of 44 PDXs showed *HLA-B*, *TAP1* and *TAP2* transcript levels below or within the same range as the lowest values of normal tissues (Br and Ts), indicating that transcriptional downregulation at these loci is prevalent among pediatric solid tumor models (**Figure 11**; **Supplementary Table S15**).

Among nonclassical HLA class I genes, the transcriptional levels of *HLA-E* and *-F* genes were below the levels of Br and Ts in a majority of PDXs, and were particularly low or negligible in neuroblastoma, and all but one (GR-RMS-14) rhabdomyosarcoma PDXs. However, *HLA-E* transcript levels were slightly elevated in four out of six Ewing sarcoma, all four HGG, three out of four NRSTS, and five out of eight osteosarcoma PDXs. Furthermore, *HLA-F* transcript levels were slightly above the lowest value of normal tissues (Br) in two HGG, three osteosarcoma, and two NRSTS PDXs. In most cases, coexpression of *HLA-E* and *-F* tends to occur in tumors with high transcript levels of *HLA-A*, *TAP1/2* and *B2M* genes (**Figure 11**; **Supplementary Table S15**). On the other hand, *HLA-G* transcripts were detected in control pooled placenta but not in any PDX, using a Taqman probe located at the boundary of exons 5 and 6 encoding the transmembrane and proximal cytoplasmic domains (data not shown). Therefore, *HLA-E* and *-F* but not full-length *HLA-G* are transcriptionally expressed in a fraction of PDX models of pediatric solid tumors, particularly in Ewing sarcoma and HGG, respectively, in agreement with reports in Ewing sarcoma (81) and adult gliomas (82) for *HLA-E*, and may contribute to HLA-dependent immunomodulation in these tumors.

## Discussion

Our study is the first comprehensive and pan-cancer analysis of HLA genotypes, expression, and mutational landscape in recurrent or refractory pediatric solid cancers and patient-matched PDX models. We observed heterogeneous HLA homozygosity frequencies across tumor types and subtypes and identified candidate positive and negative allele associations, some discriminating specific tumor subtypes (such as eRMS and aRMS, and neuroblastoma 11qWT and 11qLOH) and others shared across tumor types. Partial or complete loss of HLA class I expression is prominent in most tumors, ranging from ~50% of specimens in Ewing sarcoma, ~70% in osteosarcoma, ependymoma and LGG, and up to 100% in rhabdomyosarcoma, neuroblastoma, and nephroblastoma, whereas HLA-DR expression is scarce on tumor cells and rather occurs on infiltrating immune cells. Tumor somatic SNVs are uncommon in HLA and APP genes, whereas HLA LOH caused by CNVs was heterogenous depending on the tumor types and subtypes; it was globally low (< 10%) in neuroblastoma, nephroblastoma and ependymoma, but elevated in osteosarcoma and GBM for class I and II genes (up to ~24%), and in Ewing sarcoma and LGG for class II genes (up to 50%). On the other hand, HLA class I loss is frequently associated with transcriptional silencing of *HLA-B* and *TAP* genes in PDX models.

We determined HLA genotypes from paired normal and tumor WES, and from tumor RNA-Seq in 90.3% of patients, using HLA-HD and HLAProfiler, together with Optitype, xHLA and HISAT-genotype in unresolved cases. Overall, consensus genotypes were established for eight HLA loci (*A*, *B*, *C*, *DRB1*, *DQA1*, *DQB1*, *DPA1*, *DPB1*) in 97.2% of patients. We also observed congruent genotyping from germline (blood), patient tumor and PDX NGS data. Importantly, in cohorts with ≥ 20 EUR patients, all the *HLA-A-B-C* and -*DRB1-DQA1-DQB1* genotypes corresponded to combinations of known and/or variant haplotypes, differing only for one allele of a single locus at the 4-digit level which were all part of G groups (except the two DQB1*05:52 and *06:46 alleles absent in the database from ref. 70). Furthermore, we identified known *DRB1-DRB3/4/5* haplotypes in all cases, demonstrating the accuracy of HLA typing data in our study.

We observed a wide range of HLA homozygosity frequencies across tumor types and subtypes, including elevated frequencies for *HLA-A* and *-DP* in neuroblastoma with MYCN-A tumors (28.6 and 35.7%, respectively), *HLA-DP* in neuroblastoma with 11qLOH tumors (35%), and *HLA-DR* and *-DQ* in osteosarcoma (13.2% of patients), whereas *HLA-B* homozygosity was not observed in patients with HGG. Of note, the detection of a single allele was referred to as homozygosity, although constitutional uniparental isodisomy (83, 84) cannot be ruled out without pedigree analysis or SNP-based genotyping. Nevertheless, increased frequencies of patients with single HLA allele or haplotype in neuroblastoma with MYCN-A tumors and osteosarcoma could result in narrowed T cell repertoires against tumor antigens and restricted immunosurveillance capacities. In contrast, the absence of *HLA-B* homozygosity in HGG might reflect some selection pressure to ensure the occurrence of HLA-KIR interactions, possibly contributing to immune escape and tumor development. Future progress in characterizing T and NK cells will clarify whether HLA-KIR epistatic effects play a role in these tumors.

We found a number of HLA alleles either underrepresented (some not detected) or overrepresented in EUR patients in comparison with controls, which could represent candidate negative or positive HLA associations, and some discriminated specific tumor subtypes. For instance, the A*03:01 allele was not detected in aRMS, and the C*07:01 and *07:02 alleles were specifically underrepresented in eRMS and aRMS, respectively. Other alleles (B*07:02, DRB1*15:01, DQB1*06:02) were overrepresented in eRMS but underrepresented in aRMS. In neuroblastoma, several alleles were specifically underrepresented in 11qWT (DRB1*15:01, DQB1*06:02) or 11qLOH (DQB1*05:01, DPB1*04:02), and others were specifically overrepresented in 11qLOH (A*26:01, C*15:02, DQA1*01:04, DQB1*05:03). In HGG, the B*44:02 and DRB1*01:01 alleles were not detected and other alleles were specifically under- (B*08:01, DRB1*15:01, DQA1*01:01, DQB1*06:02) or overrepresented (DRB1*07:01, DQB1*02:02) in GBM but not in other HGG; the DQA1*01:01 allele was actually overrepresented in non-GBM. Overall, several alleles were recurrently underrepresented (B*08:01, DRB1*15:01, DQB1*06:02), supporting the existence of shared negative HLA associations. Other alleles were slightly but recurrently overrepresented (B*51:01, B*18:01, C*12:03), possibly reflecting shared positive HLA associations. Notably, the allele variations resulted in several candidate haplotype associations. For instance, the A*02:01-B*44:02-C*05:01 haplotype was overrepresented particularly in three cohorts (neuroblastoma MYCN-A, ependymoma, aRMS), whereas three *DRB1-DQA1-DQB1* haplotypes (*15:01-*01:02-*06:02, *03:01-*05:01-*02:01, and *07:01-*02:01-*02:02) showed discordant frequencies across multiple tumor types and subtypes. Therefore, specific HLA alleles and haplotypes may be negatively or positively associated to the development of pediatric solid tumors. Most patients in the MOSCATO-01 and MAPPYACTS trials had been treated by surgery and multimodal chemotherapy but not ICI prior to inclusion, ruling out that the candidate HLA associations could be linked primarily to immunotherapy outcome. It is plausible that these HLA alleles differ in their capacity to present tumor-derived peptides to T cells, and possibly to interact with receptors such as KIRs, LILRBs and CD94/NKG2 on NK and T cells, thereby differentially regulating antitumor responses. Underrepresented HLA alleles could be associated with effective T responses against cancer driver mutations, and thus individuals carrying these alleles may have a reduced risk to develop such tumors. Conversely, overrepresented HLA alleles could be associated with inefficient or unfavorable responses facilitating tumor progression and/or relapse. Our observations support a hypothesis proposed from the analysis of HLA alleles and neoantigens in The Cancer Genome Atlas (TCGA), which correlates the underrepresentation of HLA alleles capable of strong binding of neoantigen-derived epitopes to the magnitude of immunoediting in patients (85). Accordingly, the interplay with specific mutational patterns could explain subtype-dependent candidate HLA associations. For instance, eRMS and aRMS have different genetic alteration profiles (with frequent *TP53* loss and activating mutations in the RAS pathway in eRMS, and recurrent chromosomal translocations generating PAX3 or PAX7::FOXO1 fusion proteins in aRMS) and immune contexture (with a higher degree of immune infiltration in eRMS) (86). On the other hand, neuroblastoma 11qLOH tumors are associated with higher disease stage and poor outcome and exhibit a more immunosuppressive TME than 11qWT tumors (87, 88). Characterization of the HLA peptidome in relation with allele frequencies and tumor mutational profiles will allow to clarify the range of immunoediting across pediatric tumors. Of note, the patients in our study were from Europe (mostly France), whereas the reference allele and haplotype frequencies for EUR/CAU individuals were established from the US population (70, 71, 76, 77), which could explain the increased occurrence of some variant alleles and haplotypes, and possibly slightly affect frequency comparisons. We did not investigate HLA allele frequencies by gender and between primary refractory/recurrent and metastatic disease, since the limited numbers of cases would also preclude statistical analysis. HLA genotyping in additional independent cohorts are needed to demonstrate the existence of *bona fide* risk or protective HLA alleles in pediatric cancers, and to better understand their correlation with tumor immunogenicity.

Of note, a study in osteosarcoma (with > 90% of primary diagnosis tumor cases) reported putative HLA associations, with several class II alleles claimed to be negatively (DRB1*03:01, DQB1*02:01) or positively (DQA1*01:01) associated with disease risk; however, only odd ratio values were described but not allele frequencies (89). Furthermore, a genome-wide association study in one cohort did not identify a susceptibility locus within the HLA (90). Our study only identified DQB1*06:02 as a candidate protective allele in osteosarcoma. Further studies with independent cohorts, and possibly patient stratification based on specific immunological or molecular subtypes, are required to demonstrate the significance of candidate HLA association(s) in this tumor type.

We analyzed HLA class I expression by IHC (using the EMR8-5 antibody directed against a monomorphic HLA-A, -B and -C epitope) in 188 tumor specimens from 10 tumor types. The frequencies of partial or complete HLA class I antigen loss (score 0-2) ranged from 50 to 100% of tumors, corroborating previous studies in selected pediatric solid cancers by IF or IHC using various methods and reagents (52–59), and are comparable to those observed in adult solid cancers (44). Therefore, advanced pediatric and adult solid tumors frequently exhibit common patterns of altered HLA class I expression to avoid T cell-dependent immune recognition. On the other hand, the absence or low and focal HLA class II expression by tumor cells implies that the HLA class II antigen presentation pathway in pediatric solid cancers occurs primarily through the TME, such as infiltrating myeloid and/or B cells, and the immunological and therapeutic implications require further investigations.

The analysis of somatic SNVs and CNVs revealed that only few tumors harbored mutations in HLA and APP genes, while LOH due to CNVs in HLA class I and II genes occur in 5.9 to 16.7% of all cases (in *HLA-A* and *HLA–DQA1*, respectively); it reached 16.9 to 21.1% in osteosarcoma and GBM for HLA class I genes, and 20.9 to 35.7% in Ewing sarcoma and LGG for class II (*DRB1-DQA1-DQB1*) genes. A large-scale analysis of 59 adult cancers, using an allele-specific CNV calling method, revealed a prevalence of HLA class I LOH ranging from about 2 to 42% depending on the tumor type (~11% in sarcomas), and 85% of LOH involved all three *HLA-A*, *-B* and *-C* genes (49). A study of 15 adult cancers using an allele-specific machine learning algorithm reported a prevalence of HLA class I LOH ranging from 4 to 40% (in liver cancer and HNSCC, respectively), with 76% of LOH involving all three *HLA-A*, *-B* and *-C* genes, and 73% had a size > 1 Mb. The Sequenza pipeline had ~93% specificity and 95% sensitivity to detect a deletion in HLA genes (50). Recently, another pan-cancer analysis of 58 tumor types reported HLA class I LOH in 17% of primary and 18% of metastatic tumors; the prevalence of HLA class I LOH was ~22% in primary osteosarcoma, and ~15% in metastatic sarcoma tumors (51). In our study, the majority of LOH events detected in the HLA region using Sequenza (and the hg19 genome assembly) had size ranging from 50 kb (considered as a threshold for analysis) to ~32 Mb and contained multiple genes, including many with limited polymorphism, and therefore likely represent a reasonable estimate of HLA LOH prevalence in advanced pediatric solid cancers. Studies in adult cancers have focused primarily on HLA class I LOH (46–51). However, our data support a higher prevalence of HLA class II LOH in a significant fraction of pediatric sarcoma and CNS tumors. It should be noted that the smallest CNV events above the analysis threshold (≥ 50 kb) in the HLA region contained the *DRB1-DQA1-DQB1* genes, often without other annotated element, a region possibly proned to generate false positive calls from WES data due to limited sequence coverage in conjonction with allelic divergence from the reference hg19 assembly (91). Notably, HLA class I and II LOH detected in two osteosarcoma and one HGG tumors were confirmed in matched PDX models by HLA typing from both PDX WES and RNA-Seq. All three cases harbored two large CN-LOH events (in the *A-C-B-*class III-*DRA*, and *DQB1-DP* or *DRB1-DQ-DP* regions), and the two osteosarcoma tumors also had small LOH or CN-LOH in *DRB1-DQA1-DQB1* (81 kb), or *DRB1-DQA1* (48 kb) and *DQA1-DQB1* (28 kb) (data not shown). Worth mentioning, highly focal LOH events (< 50 kb) identified using Sequenza (representing ~35% of CNVs and excluded from the current analysis) were more frequent in *HLA-A* than *-B* and *-C* and recurrently occurred in the polymorphic exons 2 and 3, but had comparable prevalence in *HLA-DRB1, -DQA1, -DQB1* and *-DPB1* and more often occurred in the downstream exons, particularly in *HLA-DPB1* exons 5 and 6 encoding the transmembrane and cytoplasmic domains. Sequence divergence analysis between alleles from patients and hg19, in groups with or without LOH, did not reveal clustering of divergent alleles which could reflect alignment biases (data not shown). Whether these putative focal CNVs represent true alterations, possibly leading to the expression of truncated soluble HLA class II ectodomains by tumor cells and impairment of CD4+ T cell responses, deserve further investigation. One limitation of our CNV analysis using a reference genome assembly is the inability to detect the lost HLA alleles, and patient allele-specific methods (46, 48–50) must be implemented in precision medicine trials. We did not investigate CNVs occurring in the absence of LOH, although gain or amplification of *HLA*, *B2M* and *CIITA* genes, and possibly copy number imbalance across loci, may have biological consequences. We also explored SNVs in the coding sequences of HLA, APP and KIR genes. There was no bias of mutation rates in HLA class I and II in comparison with APP genes, and only a limited number of mutations in the highly polymorphic KIR genes, suggesting that the variant calling method is not biased by the genetic complexity of these regions. We did not investigate somatic variations in noncoding sequences, including promoters and enhancers, and cannot rule out the existence of rare alleles with polymorphism in regulatory regions which could affect HLA expression in some patients.

Structural variations are frequent in pediatric solid tumors, particularly in osteosarcoma, adrenocortical carcinoma, H3K27M-mutated HGG, and Sonic hedgehog (SHH)-activated medulloblastoma, and may result from diverse mechanisms, including chromothripsis and chromoplexy. They can lead to inter- or intrachromosomal translocations, and eventually generate recurrent oncogenic gene fusions such as *EWSR1::FLI1* in Ewing sarcoma and *PAX3* or *PAX7::FOXO1* in fusion-positive rhabdomyosarcoma (1, 3). Putative translocations in *HLA*, *B2M* or *CIITA* genes were not investigated in our study. However, recurrent gene fusions in pediatric cancers rarely involve these regions, with only two cases validated in 48 tumors from the MOSCATO-01 cohort (two translocations t (6, 6) and (6, 17) generating *GPANK1*::*ABHD16A* and *PPP1R18::FXR2* gene fusions in eRMS and osteosarcoma cases, respectively) (3, 60). Whether translocations may occur in these regions, and eventually affect regulatory domains such as enhancers, promoters, or 3’ untranslated regions, remain to be established.

We observed that HLA class I loss was associated in most cases with low transcriptional levels of HLA class I, *TAP1/2* and *B2M* across several tumor types, by RT-qPCR (Taqman assay) using a panel of PDXs, particularly in neuroblastoma, rhabdomyosarcoma, and nephroblastoma. In neuroblastoma and small-cell lung cancer, the coordinated transcriptional silencing of components of the MHC class I antigen presentation pathway (including MHC class I heavy chains, *TAP1/2*, *PSMB8/9*, and *NLRC5* transactivator) is associated with activity of the polycomb repressive complex 2 (PRC2) and bivalent H3K4me3 and H3K27me3 histone modifications. Pharmacological inhibition of the PRC2 catalytic (enhancer of zeste homolog 2, EZH2) or regulatory subunit (embryonic ectoderm development, EED) synergizes with IFNγ to upregulate MHC class I expression and cell surface levels, and overcomes resistance to T cell killing *in vitro* (92). Neuroblastoma plasticity defines two adrenergic and mesenchymal epigenetic cell states associated with inactive or active responsiveness to inflammatory sensing, including HLA class I upregulation by the TLR3-activating double-stranded (ds)RNA mimetic poly (I:C) (93), and immune response genes are indeed epigenetically silenced by the PRC2 complex in adrenergic tumor cells (94). Another study showed that the double homeobox 4 (DUX4) protein is expressed in a wide range of adult solid cancers, including soft-tissue sarcomas, and blocks IFNγ-mediated MHC class I induction in tumor cell lines and immortalized myoblasts (95). Thus, distinct mechanisms may underlie HLA class I silencing across pediatric solid tumors such as neuroblastoma and rhabdomyosarcoma. It will be also interesting to investigate whether DUX4 similarly mediates HLA class I silencing in *CIC::DUX4* gene fusion positive sarcomas. Notably, *HLA-B*, *TAP1* and *TAP2* transcript levels were lower than the minimum values of control normal tissues (brain and testis) in ~80% of PDXs (32 out of 44), and *B2M* levels were also low in neuroblastoma, nephroblastoma, and all but one rhabdomyosarcoma tumors. Furthermore, three out of four Ewing sarcoma, two osteosarcoma, and single eRMS, aRMS, and HGG PDXs with high HLA class I IHC score showed low or negligible *HLA-B* transcript levels. These observations suggest that *HLA-B* silencing is more prevalent than global HLA class I loss revealed using the EMR8-5 antibody. HLA class I and APP loss in Ewing sarcoma cell lines is reversible using IFNγ (56). Other studies in adult tumors showed that inhibitors of epigenetic modifiers such as histone deacetylases (HDACs) (96) and lysine-specific histone demethyase 1A (KDM1A/LSD1) (97) can also restore or upregulate HLA class I expression and antigen presentation. Indeed, the HDAC inhibitor entinostat was recently shown to increase neuroblastoma immunogenicity *in vitro*, resulting in increased T and NK cell cytotoxicity, accompanied by an adrenergic-to-mesenchymal cell lineage shift (98). A systematic evaluation of immunostimulatory factors upregulating HLA class I expression across pediatric tumor types is warranted for clinical prioritization.

Interestingly, in HGG, we observed a high HLA class I IHC score in three out of four PDX models, in agreement with a study of patient-derived orthotopic xenograft (PDOX) models by flow cytometry, which showed higher levels of cell surface HLA class I antigens in HGG than other pediatric brain tumors including medulloblastoma and ependymoma (99). However, only four out of 21 (19%) patient tumor samples showed a high HLA class I IHC score. It is possible that tumor-extrinsic mechanism(s) related to immune pressure contribute to a higher frequency of HLA class I loss in patients, and is alleviated upon propagation in immunodeficient (NSG) mice. In addition, by HLA typing from RNA-Seq, we detected a pattern consistent with LOH in one giant cell GBM PDX, which was associated with low *HLA-B* transcript levels. Further work is needed to clarify the extent of locus-specific HLA alterations in HGG, and possibly the differential HLA class I expression in patient tumors and PDXs.

We detected low but consistent *HLA-E* transcript levels in all four HGG and ~60 to 75% of Ewing sarcoma, osteosarcoma, and NRSTS PDXs. We also detected *HLA-F* transcript levels slightly higher than the lowest value of normal tissues (brain) in ~40 to 50% of HGG, osteosarcoma, and NRSTS PDXs. Overall, *HLA-E* and *-F* coexpression correlated with high levels of *HLA-A*, *TAP1/2* and *B2M* transcripts across tumor types. The HLA-E antigen present peptides derived from the leader sequences of HLA-A, -B, -C and -G to inhibitory NKG2A/B or activating NKG2C receptors paired with CD94 and expressed on NK and CD8+ T cells; self- and pathogen-specific HLA-E-restricted cytotoxic CD8+ T cells have been also detected (100). In Ewing sarcoma, a recent IHC study has reported that 22 out of 26 (85%) pre-treatment tumors were positive for HLA-E expression on tumor cells, infiltrating macrophages, or both; however, HLA-E expression on tumor cells did not impair the preclinical activity of GD2-specific chimeric antigen receptor (CAR) T cells (81). HLA-E expression has been also reported in adult astrocytoma and GBM, being particularly prominent in pseudopalisades associated with tumor invasion, and protects glioma cells from NKG2D-mediated lysis by NK cells *in vitro* (82). On the other hand, the HLA-F antigen can exist as both an empty conformer lacking peptide and β2m and a peptide-presenting molecule, which are ligands for LILRBs and KIRs (101). Whether HLA-E and HLA-F expression promotes immune evasion from NK and T cells or contribute to expand the HLA repertoire on tumor cells remains to be established. Of note, HLA-G expression is induced by IFNγ in Ewing sarcoma cell lines, and is detected in a majority of tumor biopsies on both tumor and infiltrating myeloid cells (81, 102). In our study, we did not detect *HLA-G* expression in any PDX by Taqman assay targeting the exon 5-6 boundary present in transcripts encoding membrane-bound HLA-G isoforms, in accordance with an inducible rather than constitutive expression. Furthermore, *HLA-G* transcripts were rarely detected from patient tumor RNA-Seq using HLA-HD or HLAProfiler, with the exception of all four alveolar soft part sarcoma tumors (data not shown).

It is tempting to speculate that specific HLA genotypes may negatively impact early immune surveillance in pediatric cancers, whereas HLA class I and APP antigen loss, mostly through transcriptional silencing, further contribute to tumor progression, relapse and/or dissemination. These observations have several implications. First, ongoing efforts to identify HLA-restricted T cell epitopes from tumor antigens in pediatric cancers need to prioritize the most frequent HLA alleles in specific cancer types and subtypes, which are not necessarily the most frequent in the general population. Second, the scarcity of tumor somatic mutations in HLA and APP genes contrasting with their frequent transcriptional silencing in most advanced pediatric solid tumors suggest that restoration or upregulation of HLA expression might be achievable through epigenetic modulation in at least some of the patients. However, such strategies could remain inefficient as monotherapy in patients with unfavorable HLA genotypes, because of their restricted capacities to mount efficient antitumor T cell responses, and it should be evaluated whether pharmacological approaches can provide other immunostimulatory benefit in these individuals (e.g. immunoproteasome induction and/or modification of the HLA peptidome by IFNγ and epigenetic modifiers). Third, patients with irreversible HLA alterations such as LOH might be less likely to benefit from T-cell immunotherapy approaches, and must be identified prior to inclusion into such clinical trials. Importantly, pediatric patients with solid tumors have reduced numbers of circulating naïve T cells at diagnosis, particularly in rhabdomyosarcoma, Ewing sarcoma, neuroblastoma and nephroblastoma (103). This could contribute to the failure of tumor immunosurveillance, and limit the development of tumor-specific T cell responses despite increasing tumor immunogenicity, and it will be critical to elucidate the underlying mechanisms. Nevertheless, T cell recognition exhibit exquisite sensitivity, and as few as a single HLA class I-peptide complex can elicit cytotoxicity toward target cells (104). Recently, HLA-restricted PHOXB2 CARs have been developed against neuroblastoma tumors, and were shown to mediate specific killing *in vitro* and complete tumor regression in mice despite very low tumor HLA class I antigen expression (105). Because of the frequent *HLA-B* and *TAP* transcriptional silencing in pediatric tumors, the identification of TAP-independent epitopes could also provide relevant T cell targets, particularly in HLA-A2-positive patients, as reported for TAP-deficient adult tumor cell lines (106) and in preclinical studies (107). Furthermore, targeting HLA LOH with allele-sensing bispecific CAR T cells could represent another potent strategy (108, 109). On the other hand, antigen-specific CD4+ T cells can mediate protective immunity even against MHC-II-negative tumors (110, 111), and thus characterization of the HLA class II peptidome from the TME could allow to identify additional antigenic determinants capable to mediate antitumor T cell responses. Therefore, it can be expected that the combination of patient immunogenetic and tumor molecular profiling in future clinical trials will allow to identify effective T cell-based immunotherapy strategies in a significant fraction of pediatric patients with advanced solid tumors, as in adults.

## Materials and methods

### Patients, genomic data, and PDX models

Patients with recurrent or refractory pediatric solid tumors previously enrolled in the institutional MOSCATO-01 (60) and international MAPPYACTS (61) trials (*n* = 39 and 537, respectively) were included in this study (**Table 1**; **Supplementary Table S1**). Normal and tumor WES and tumor RNA sequencing (RNA-Seq) were performed within the main studies as described (60, 61). Briefly, WES was captured from whole blood and tumor DNA using Agilent SureSelect V5 (50Mb), Clinical Research Exome (54Mb), SureSelect XT human All exon CRE, or Twist Human Core Exome Enrichment kit. For RNA-Seq, libraries were prepared from tumor poly(A) mRNA using the TruSeq Stranded mRNA kit, and sequencing performed using Illumina sequencers (NextSeq 500 or Hiseq 2000/2500/4000) in 75 bp paired-end mode (with mean depth of coverage 100x for WES in MAPPYACTS). The trials were approved by independent ethics committees, national medical authorities and conducted according to the principles of the Declaration of Helsinki. Written informed consent was signed by the patient or parents/legal representative and assent of the minor child according to local laws for the main study and for ancillary research studies. The establishment and molecular characterization of PDX models have been described elsewhere (79). Sequencing data and clinical annotations from patients and PDXs have been deposited in the European Genome-phenome Archive (Accession No. EGAS00001005935 and EGAS00001007327) (61, 79).

### HLA allelic inference

Four-digit HLA alleles for HLA class I (*HLA-A*, *-B*, *-C*) and class II (*HLA-DRB1*, *-DQA1*, *-DQB1*, *-DPA1*, *-DPB1*) genes were initially inferred from normal WES, tumor WES, and tumor RNA-Seq using HLA-HD v1.3.0 (62), and from tumor RNA-seq using both HLA-HD and HLAProfiler v1.0.0 (63). HLA typing accuracy of > 99% for *HLA-A*, *-B*, -C, *-DRB1*, and *-DQB1* have been reported for HLA-HD from high-coverage WES (62) and HLAProfiler from RNA-Seq (63). Furthermore, typing accuracy from 76.5 to > 99% for *HLA-DQA1* and 90.4 to 100% for *HLA-DPA1* and *-DPB1* have been reported for HLA-HD from WES and RNA-Seq (**Supplementary Table S2**). For each HLA gene of a patient, a consensus genotype was established when two distinct alleles were determined using both HLA-HD and HLAProfiler from two different NGS samples. When both methods identified a discordant genotype or a single allele only, typing was repeated from normal WES using Optitype v1.3.2 (*HLA-A*, *-B*, *-C*) (64), xHLA v1.2 (*HLA-A*, *-B*, *-C*, *-DRB1*, *-DQB1*, *-DPB1*) (65) and HISAT-genotype v1.3.2 (*HLA-A*, *-B*, -*C*, *-DRB1*, *-DQA1*, *-DQB1*) (66). Benchmarking studies have reported typing accuracy from 94.9 to 100% for HLA class I and 78.6 to 100% for HLA class II genes using Optitype and HISAT-genotype, while a broader range has been reported for xHLA (41.8 to > 98%) (**Supplementary Table S2**). A final consensus genotype was established for each HLA gene when at least two algorithms identified the same allele(s) from two different NGS samples. HLA typing was performed using FASTQ (HLA-HD, HLAProfiler, Optitype, HISAT-genotype), or BAM files (xHLA). HLA-HD was run with minimum read length (*-m*) of 75 and trimming option (*-c*) of 0.95. The default parameters were used for HLAProfiler and HISAT-genotype. For Optitype, the recommended parameters include a default percent identity (*-i*) of 95, maximum hit (*-m*) of 1, distance range (*-dr*) of 0 for reads filtering using Razers, and *–dna* option for WES data. For xHLA, FASTQ files were preprocessed into BAM files using the embedded “*get-reads-alt-unmap.sh*” script before typing using default parameters.

### Genetic ancestry assignment

#### Germline variant calling

Normal WES data pre-processing and variant calling was performed using the Nf-core Sarek pipeline (v2.6.1) (112). The sequencing quality of WES data was assessed with FASTQC (v0.11.9) (113) prior to alignment to the human reference genome (GRCh38) using BWA-MEM (v0.7.17) (114) and pre-processing based on GATK best practices (v4.1.7) (115). Germline variant calling was performed using HaplotypeCaller, and gVCFs output files were aggregated per tumor type using CombineGVCFs. Joint genotyping of aggregated gVCFs was performed using GenotypeGVCFs, and variant recalibration was performed using VariantRecalibrator and ApplyVQSR *(–truth-sensitivity-filter-level 99.9)* as implemented in the GATK workflow (115). Variants were filtered using VCFtools (v0.1.16) (116) using the option *–remove-filtered-all*, to include only high quality SNPs (PASS filter).

#### Genetic ancestry annotation using EthSEQ

Genetic ancestry was computed from patient normal WES using EthSEQ (v2.1.4) (67, 68). Genotype data of 1,549 individuals from the 1000 Genomes Project phase 3 release (117), representing the five major superpopulations (AFR, AMR, EAS, EUR, SAS), were used to build a reference model for ancestry annotation. The reference model includes SNPs with minor allele frequency (MAF) > 1% present in the captured regions from the Agilent SureSelect, Clinical Research Exome, SureSelect XT human All exon CRE, and Twist Human Core Exome Enrichment kits. Pre-processed VCF files containing genotype data of patients were combined for each tumor cohort to build target models. The SNPs common between the reference and target models were selected by EthSEQ to create an aggregated model for principal component analysis (PCA), and ancestry annotations inferred by EthSEQ from 3D PCA spaces comprising the first three principal components. Multistep refinement analysis mode was performed to improve the ancestry characterization of spatially-close populations, such as EUR and AMR. Admixture estimates were plotted for all patients per tumor type using the barplot function in R (118).

### HLA haplotype assignment

*HLA-A-B-C and -DRB1-DQA1-DQB1* haplotypes were inferred using EM-estimated frequencies of known haplotypes identified in EUR donors from the NMDP (70, 71). When one putative haplotype diverged from a reference haplotype by a single allele at 4-digit resolution, it was considered as a variant haplotype and assigned to the closest haplotype number with addition of a suffix (b, c, d, etc.). Patients with genotypes containing at least two divergent HLA alleles from the reference haplotype(s) were considered to have unknown haplotypes. *HLA-DRB1* (DR1, DR8), *-DRB1-DRB3* (DR52), *-DRB1-DRB4* (DR53), or -*DRB1-DRB5* (DR51) haplotypes were inferred according to known haplotype combinations (72–74) (**Supplementary Table S7A**).

### HLA allele frequencies in EUR patients and controls

Patients with an EUR ancestry fraction ≥ 70% determined by EthSEQ were combined to calculate mean HLA allele frequencies for each cohort. Mean allele frequencies were calculated by dividing the number of occurrence(s) of a given allele by the number of chromosomes in the study population. As references, mean HLA class I allele frequencies were calculated using data reported in individuals from the NMDP who self-reported as “European Caucasian” (EURCAU, *n* = 1,242,890 for *HLA-A* and *-B*, and *n* = 395,676 for *HLA-C*) (70), and in a study of individuals with self-reported European ancestry from the USA (EURA, *n* = 2,248) (76). For HLA class II genes, mean allele frequencies were calculated using data reported in a study of self-reported EURA individuals from the NMDP (*n* = 1,899) (71), those in the aforementioned references (70, 76), and in a study of organ transplant recipients and CAU donors at the Loyola University Medical Center for *HLA-DPB1* (77) (**Supplementary Tables S9A, B**).

### Immunochemistry

An indirect immunoperoxidase technique was applied to 4-µm-thick deparaffinized sections of formalin-fixed paraffin-embedded tumor tissue samples, using an automate stainer (Benchmark Ultra, Ventana, Tucson, AZ). Primary antibodies were the mouse monoclonal antibody EMR8-5 directed to HLA class I (HLA-A, -B, -C) (MBL Life Science, Woburn, MA) (final concentration 0.5 µg/mL), and a rabbit polyclonal antibody anti-HLA-DRB1-3 (LSBio, Lynnwood, WA) (final concentration 3.3 µg/mL). All stained sections were interpreted by two experienced pathologists (NL, JYS). Expression of HLA molecules on tumor cells was provided as h-score. Internal controls were stromal cells and immune cells.

### Analysis of tumor somatic mutations in HLA and APP genes

Somatic tumor mutations were analyzed in 78 genes involved in HLA antigen processing and presentation as annotated in the Kyoto Encyclopedia of Genes and Genomes (KEGG) database (pathway hsa04612). Somatic variant calling was performed using the Illumina DRAGEN Bio-IT Platform v3.10 pipeline. Sequencing reads were first aligned to the reference hg38 genome, and somatic mutations were called in comparison between normal and tumor WES. Specimens with tumor cellularity ≥ 0.20 (as determined using Sequenza, see below) were analyzed. High quality variants in the targeted regions which passed the quality filters were further extracted based on the following criteria: (i) less than 4 reads and fraction of mutated reads < 0.06 in normal samples, and (ii) more than 3 reads and fraction of mutated reads ≥ 0.15 in tumor samples.

### CNV analysis

CNVs localized in the chromosome arms 6p, 15q and 16p were inferred from patient paired normal and tumor WES data, in BAM format, using the Sequenza pipeline (78) and the reference genome assembly GRCh37 (hg19). Positions were mapped to GRCh38 using the LiftOver tool from the University of California Santa Cruz (UCSC) Genome Browser (119). Samples with tumor cellularity ≥ 0.2 and CNVs with a minimal size ≥ 50 kb, containing at least one targeted gene annotated in the Ensembl database release 109 (120), were retained. The Sequenza pangenomic profiles were manually inspected to assess aneuploidy states. Gene content was extracted from Ensembl using the R/Bioconductor biomaRt package (121). LOH was determined by the loss of minor allele and CNA = CNt = 1. CN-LOH was determined by CNA = CNt = 2, under the reductionist assumption that two copies of the remaining allele define a minimal CN-LOH event compared to germline, independently of aneuploidy. LOH with co-occuring gain were defined by CNA = CNt ≥ 3, and LOH with amplifications when CNt ≥ 7 if ploidy = 2, or CNt ≥ (1 + twice of ploidy) if ploidy ≥ 3 and ≤ 9. Biallelic losses were identifed when CNt was zero. To calculate LOH prevalence at individual loci, all events (LOH, CN-LOH, LOH+gain and LOH+amplification) were taken into account for HLA genes, whereas only LOH and biallelic losses were considered for *B2M* and *CIITA* genes.

### Real-time quantitative PCR analysis

Total RNA were extracted from PDX samples using TRIzol (Invitrogen), dissolved in DNase/RNase-free water, and quantified using the NanodropOne spectrophotometer (ThermoFisher Scientific); RNA integrity was verified using the Agilent 2100 bioanalyzer with the RNA 6000 Nano kit (Agilent Technologies). TaqMan assays (ThermoFisher Scientific) were selected for the 9 target genes and 6 reference housekeeping genes, and commercially available RNA from normal tissues (spleen, lymph nodes, brain, testis, placenta) were used as controls (**Supplementary Tables S14A, B**). The ezDNase enzyme (Invitrogen) was used to remove possible gDNA contamination from template RNA prior to the RT reaction. 500 ng of total RNA was reverse transcribed in a 20 µL final reaction volume using the SuperScript™ IV VILO™ Master Mix (Invitrogen) in the presence of RNase inhibitor. cDNA synthesis was primed with oligo(dT)18 and random hexamer primers following the manufacturer’s instructions. Quantitative PCR experiments were performed with the QuantStudio 12K Flex Real-Time PCR System (Life Technologies). For qPCR reactions, 3 ng of cDNA were mixed with TaqMan FastAdvance Master Mix and TaqMan assay in a final volume of 10 µl, loaded on 384-well microplates and submitted to 40 cycles of PCR (50°C/2 min; (95°C/1 sec; 60°C/20 sec) x40). Each sample measurement was made in duplicate, and Ct values determined for analysis. The five most stable reference genes were selected by GenEx software (MultiD), and their geometric mean used to normalize the data. The amplification efficiency of the qPCR reaction was measured for each TaqMan assay using a standard curve, prepared by a 5-fold dilution series of a pool of the three cDNAs obtained with lymph node samples. The known quantity of cDNA in the standard curve from 9 ng to 5.7 10^-4^ ng allowed to calculate the slope and the efficiency for each target. Quantification of each target gene in the samples was determined by comparing its expression level against the mean of the expression levels of the five reference genes. Target quantities were calculated using the 2^ΔCt^ method.

## Data availability statement

The data presented in the study have been deposited in the European Genome-phenome Archive, accession numbers EGAS00001005935 and EGAS00001007327 (61, 79).

## Ethics statement

The studies involving humans were approved by the Comite De Protection Des Personnes Ile De France III, Hôpital Tarnier-Cochin, 89 rue d’Assas 75006 Paris, and Gustave Roussy Institutional Review Board. The studies were conducted in accordance with the local legislation and institutional requirements. Written informed consent for participation in this study was provided by the participants’ legal guardians/next of kin. The animal study was approved by the France Ministry of Agriculture, and Gustave Roussy CEEA26 (CEEA PdL N°6, approval number: 2015032614359689 V7, 1281.01, C75-05-18, 2012-017). The study was conducted in accordance with the local legislation and institutional requirements.

## Author contributions

WL: Data curation, Formal Analysis, Investigation, Methodology, Software, Validation, Visualization, Writing – original draft, Writing – review & editing. MM: Investigation, Resources, Writing – review & editing. KG: Formal Analysis, Investigation, Methodology, Writing – review & editing. EJ: Validation, Formal Analysis, Investigation, Methodology, Resources, Writing – review & editing. LG: Formal Analysis, Investigation, Methodology, Resources, Software, Writing – review & editing. WR: Formal Analysis, Investigation, Methodology, Software, Writing – review & editing. AM: Methodology, Software, Writing - review & editing. NN: Formal Analysis, Investigation, Methodology, Writing – review & editing. DD: Methodology, Software, Writing – review & editing. MV: Formal Analysis, Investigation, Methodology, Writing – review & editing. NL: Formal Analysis, Investigation, Methodology, Writing – review & editing. AK: Investigation, Methodology, Writing – review & editing. PD: Investigation, Methodology, Writing – review & editing, Data curation. AR: Methodology, Software, Writing – review & editing. CP: Investigation, Validation, Writing – review & editing. GS: Funding acquisition, Investigation, Methodology, Resources, Validation, Writing – review & editing. MC: Investigation, Methodology, Validation, Writing – review & editing. LZ: Investigation, Resources, Validation, Writing – review & editing. J-YS: Formal Analysis, Investigation, Methodology, Resources, Validation, Writing – review & editing. BG: Conceptualization, Funding acquisition, Investigation, Methodology, Project administration, Resources, Supervision, Validation, Writing – original draft, Writing – review & editing. JS: Conceptualization, Data curation, Formal Analysis, Investigation, Methodology, Supervision, Validation, Visualization, Writing – original draft, Writing – review & editing.

## Supplementary material

Supplementary Figure 1 Spearman’s correlations between the No. of alleles and No. of patients for HLA class I **(A)** and class II genes **(B)** in cohorts with advanced pediatric solid tumors.

Supplementary Figure 2 Genetic ancestry assignment using EthSEQ in patients with eRMS **(A)**, aRMS **(B)**, osteosarcoma **(C)**, Ewing sarcoma **(D)**, NRSTS **(E)**, neuroblastoma **(F)**, nephroblastoma **(G)**, carcinoma **(H)**, LGG **(I)**, HGG **(J)**, medulloblastoma **(K)**, and ependymoma **(L)**. For each cohort, EthSEQ report represent the 2-dimensional space builts with PCA components 1 and 2 (I), 2 and 3 (II), 1 and 3 (III), and 3-dimensional space built of PCA 1 to 3 combined (IV). Polygons represent reference superpopulations (AFR, AMR, EAS, EUR, SAS), and dots represent patients from the corresponding tumor type and/or subtype.
