# Supplementary figures and images for: Divergent HLA variations and heterogeneous expression but recurrent HLA loss-of- heterozygosity and common HLA-B and TAP transcriptional silencing across advanced pediatric solid cancers
Source: Front Immunol. 2024 Jan 22;14:1265469. doi: 10.3389/fimmu.2023.1265469 (PMC10839790; doi:10.3389/fimmu.2023.1265469)

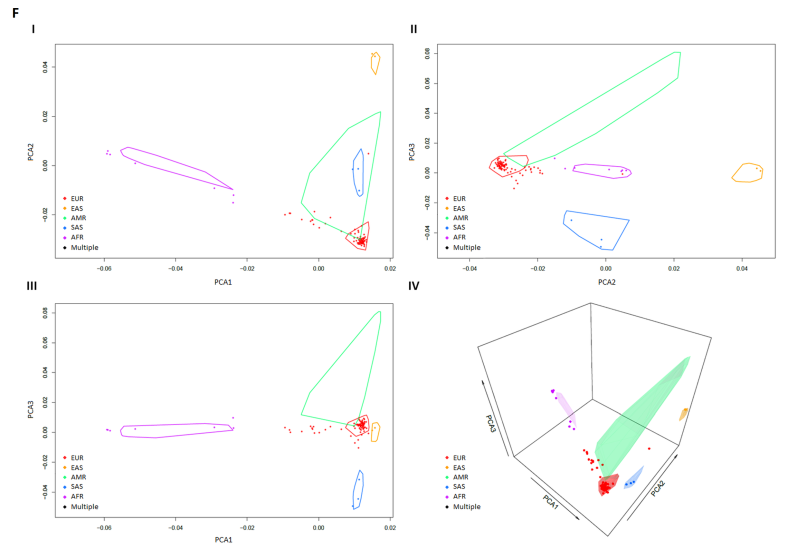

Supplement: Supplementary file 1 [file Image_3.tiff]

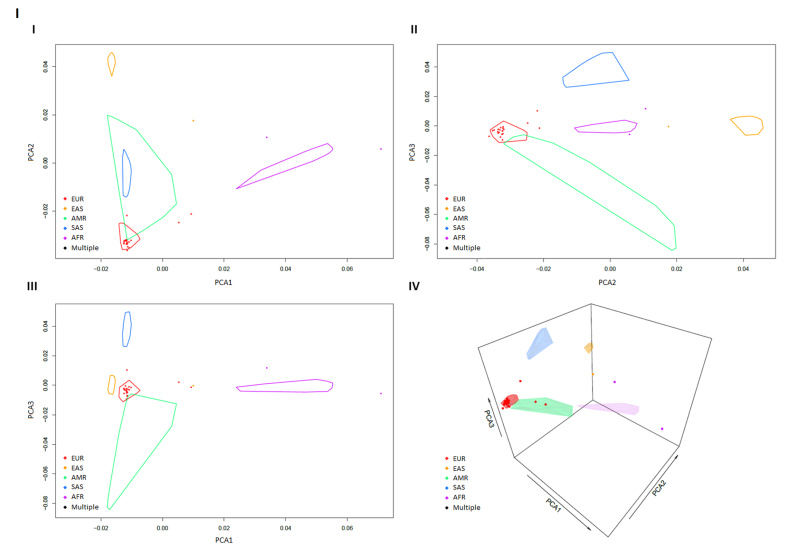

Supplement: Supplementary file 2 [file Image_4.tiff]

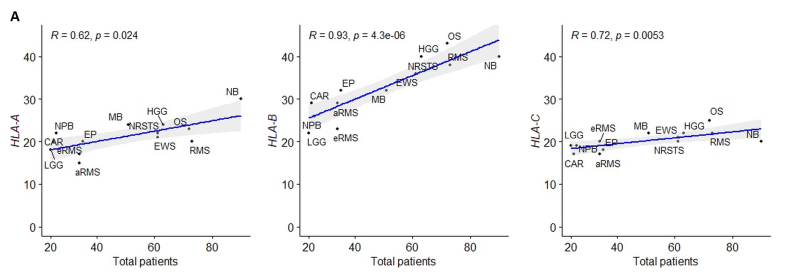

Supplement: Supplementary Figure 1 — Spearman’s correlations between the No. of alleles and No. of patients for HLA class I (A) and class II genes (B) in cohorts with advanced pediatric solid tumors. [file Image_1.tiff]

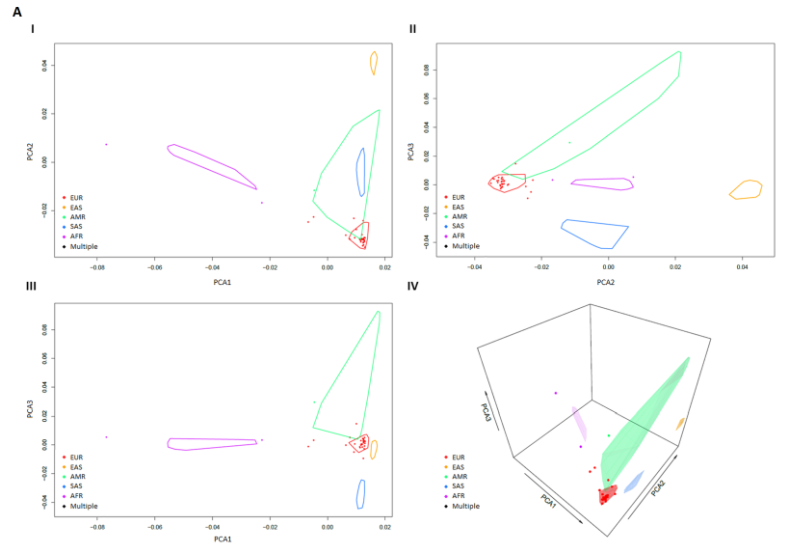

Supplement: Supplementary Figure 2 — Genetic ancestry assignment using EthSEQ in patients with eRMS (A), aRMS (B), osteosarcoma (C), Ewing sarcoma (D), NRSTS (E), neuroblastoma (F), nephroblastoma (G), carcinoma (H), LGG (I), HGG (J), medulloblastoma (K), and ependymoma (L). For each cohort, EthSEQ report represent the 2-dimensional space builts with PCA components 1 and 2 (I), 2 and 3 (II), 1 and 3 (III), and 3-dimensional space built of PCA 1 to 3 combined (IV). Polygons represent reference superpopulations (AFR, AMR, EAS, EUR, SAS), and dots represent patients from the corresponding tumor type and/or subtype. [file Image_2.tiff]
